# Transient Structure Associated with the Spindle Pole Body Directs Meiotic Microtubule Reorganization in *S. pombe*

**DOI:** 10.1016/j.cub.2012.02.042

**Published:** 2012-04-10

**Authors:** Charlotta Funaya, Shivanthi Samarasinghe, Sabine Pruggnaller, Midori Ohta, Yvonne Connolly, Jan Müller, Hiroshi Murakami, Agnes Grallert, Masayuki Yamamoto, Duncan Smith, Claude Antony, Kayoko Tanaka

**Affiliations:** 1 Electron Microscopy Core Facility, European Molecular Biology Laboratory, 69117 Heidelberg, Germany; 2 Department of Biochemistry, University of Leicester, Henry Wellcome Building, Lancaster Road, Leicester LE1 9HN, UK; 3 Department of Biophysics and Biochemistry, Graduate School of Science, University of Tokyo, Tokyo 113-0033, Japan; 4 Biological Mass Spectrometry Facility, Cancer Research UK, Paterson Institute for Cancer Research, University of Manchester, Manchester M20 4BX, UK; 5 Cell Division Group, Cancer Research UK, Paterson Institute for Cancer Research, University of Manchester, Manchester M20 4BX, UK; 6 Department of Regulatory Biology, Graduate School of Science and Engineering, Saitama University, Saitama 338-8570, Japan

## Abstract

**Background:**

Vigorous chromosome movements driven by cytoskeletal assemblies are a widely conserved feature of sexual differentiation to facilitate meiotic recombination. In fission yeast, this process involves the dramatic conversion of arrays of cytoplasmic microtubules (MTs), generated from multiple MT organizing centers (MTOCs), into a single radial MT (rMT) array associated with the spindle pole body (SPB), the major MTOC during meiotic prophase. The rMT is then dissolved upon the onset of meiosis I when a bipolar spindle emerges to conduct chromosome segregation. Structural features and molecular mechanisms that govern these dynamic MT rearrangements are poorly understood.

**Results:**

Electron tomography of the SPBs showed that the rMT emanates from a newly recognized amorphous structure, which we term the rMTOC. The rMTOC, which resides at the cytoplasmic side of the SPB, is highly enriched in γ-tubulin reminiscent of the pericentriolar material of higher eukaryotic centrosomes. Formation of the rMTOC depends on Hrs1/Mcp6, a meiosis-specific SPB component that is located at the rMTOC. At the onset of meiosis I, Hrs1/Mcp6 is subject to strict downregulation by both proteasome-dependent degradation and phosphorylation leading to complete inactivation of the rMTOC. This ensures rMT dissolution and bipolar spindle formation.

**Conclusions:**

Our study reveals the molecular basis for the transient generation of a novel MTOC, which triggers a program of MT rearrangement that is required for meiotic differentiation.

## Graphical abstract

**Graphical Abstract:**

**Figure:**
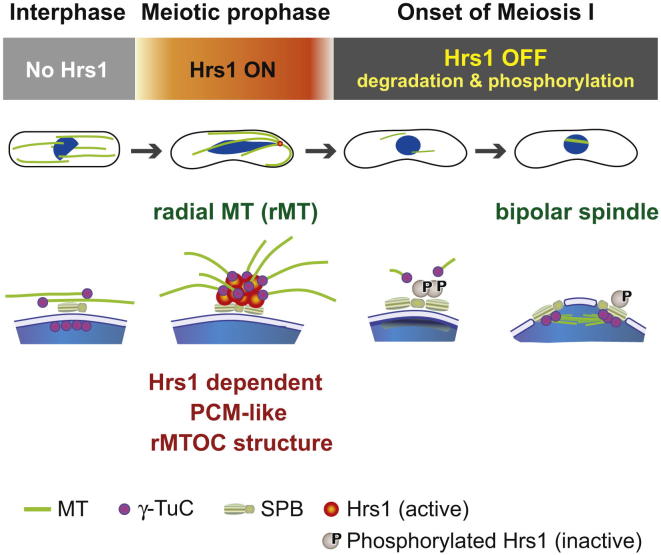

**Highlights:**

► A novel specialized MTOC (rMTOC) organizes a meiotic radial microtubule (rMT) array ► rMTOC is transiently generated to reorganize MTs appropriate for differentiation ► Hrs1/Mcp6, a potential NudE ortholog, scaffolds the rMTOC ► Both phosphorylation and degradation of Hrs1/Mcp6 ensure rMTOC inactivation

## Introduction

Microtubule organizing centers (MTOCs) play a pivotal role in defining microtubule (MT) architecture and accurate regulation of MT arrangement is crucial in both proliferation and differentiation stages. Among the most striking instances of dynamic MT reorganization are those that accompany sexual differentiation. This process is particularly well studied in fission yeast, in which numerous key MTOC components and regulators have been identified [1–5]. The fission yeast serves as an excellent model system to study a case of stage-specific MT reorganization during cell differentiation.

Vigorous chromosome movement, a common feature of meiosis in a wide range of eukaryotes, is one of the highlights during fission yeast meiosis. Throughout meiotic prophase, chromosome oscillations traversing the entire cell, known as the “horsetail” nuclear movements, occur [6, 7]. These oscillations are set up by an intricate program of MT reorganization. Pheromone signaling triggers the conversion of interphase MT bundles into a radial MT (rMT) array emanating from the spindle pole body (SPB) [8–10]. This rMT array, aided by the dynein-dynactin complex, induces vigorous oscillations of the SPB back and forth through the cell [11–14]. Pheromone signaling also induces chromosome rearrangements that result in the bouquet conformation [15], in which centromeres move away from the SPB and telomeres cluster toward it [16]. Together, the horsetail nuclear movement and the bouquet structure facilitate pairing of homologous chromosomes, leading to efficient meiotic recombination [11, 17, 18]. At the onset of meiosis I (MI), the rMT array is replaced by the MI spindle upon which reductional chromosome segregation takes place. Assembly and disassembly of the rMTs are therefore crucial to the choreography of meiosis.

We previously identified Hrs1 (also known as Mcp6 [19]), a meiosis-specific SPB component, as an rMT organizer [19, 20]. Hrs1 interacts with the N terminus of the meiotic SPB component Kms1 [21], a KASH domain protein predicted to cross the outer nuclear membrane with its C terminus residing in the nuclear periplasm, where it interacts with the SUN domain protein Sad1 [22], whereas N terminus of Kms1 is exposed in the cytoplasm [23]. Hrs1 also interacts with the γ-tubulin complex (γ-TuC) component Alp4 [24], the γ-TuC associated factor Mto1 [2], and itself [20]. Based on these observations, we proposed that Hrs1 acts as a bridge linking the cytoplasmic side of the SPB to the minus ends of MTs to arrange the rMTs (see Figure S1A available online) [20]. However, direct evidence for this model or for any structural alteration of the SPB during meiotic prophase has been missing.

In this study, we have used highly synchronous meiotic cell cultures to study both the ultrastructure of the SPB and biochemical events during meiotic progression. We provide evidence that fission yeast form a specialized MTOC structure, which we term rMTOC, during meiotic prophase. The rMTOC is reminiscent of the higher eukaryotic pericentriolar material (PCM), a proteineous amorphous structure that surrounds the centrioles and possesses high MTOC activity by accumulating many γ-tubulin molecules [25]. Hrs1 is required for rMTOC formation and is enriched at the rMTOC site along with γ-tubulin. At the transition from meiotic prophase to MI, Hrs1 is rigorously downregulated leading to complete inactivation of rMTOC. Hence our work reveals the ultrastructural and molecular basis for the transient formation of the rMTOC that choreographs the MT-driven nuclear movements uniquely required for meiosis.

## Results

### The rMTOC Resides in the Vicinity of, But Is Distinct from, the SPB

**Figure 1:**
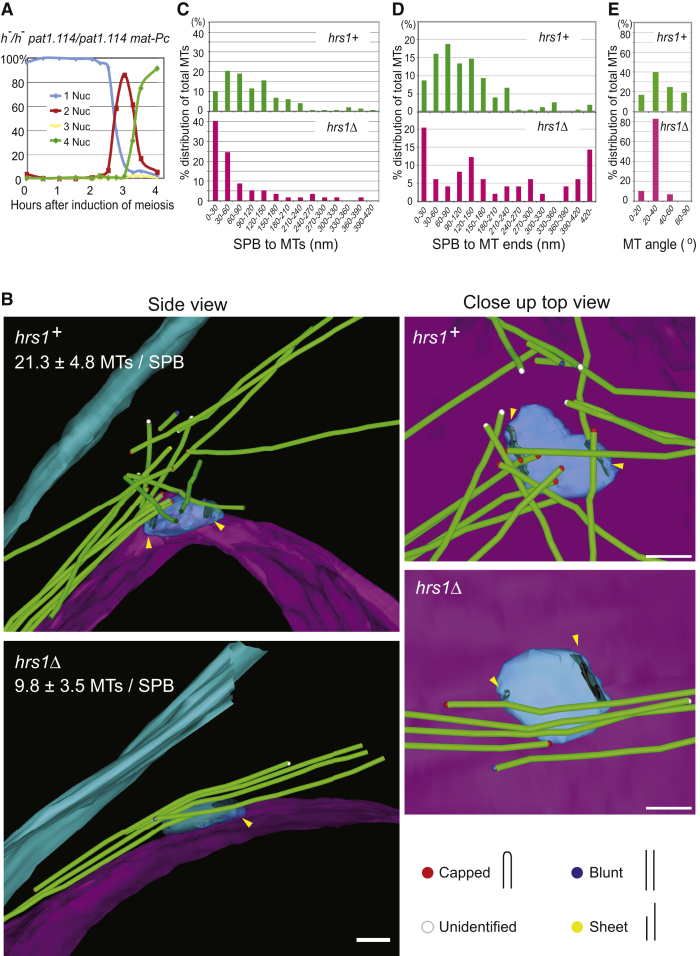
The rMTOC Resides at a Cytoplasmic Site in the Vicinity of, But Distinct from, the Core SPB (A) Highly synchronous meiosis induced with a diploid *h^−^ pat1.114 mat-Pc* strain. The *h^−^*/*h^−^ pat1.114/pat1.114 mat-Pc* strain was synchronized at G1 by nitrogen starvation before meiosis was induced by a temperature shift (see Experimental Procedures). Synchronicity was monitored by counting the number of nuclei. (B) 3D model of rMTOC arrangement. Diploid *h^−^ pat1.114 mat-Pc* cells harboring WT or deletion *hrs1* allele were induced for synchronous meiosis for 80 min (Figure S1B). They were high-pressure frozen and processed for EM-tomography analysis. In the model, MTs are green and the different colored caps indicate the MT end structure as indicated at the bottom of the panel. The SPBs are modeled in blue and lamellar bodies [41] (a laminate structure that sits next to the bridge structure of the SPB) are highlighted in black and indicated with yellow arrowheads. Both the *hrs1^+^* and *hrs1* deletion cells had duplicated SPBs with two lamellar bodies. The nuclear envelope is shown in pink, and the plasma membrane is shown in turquoise. Scale bar represents 100 nm. (C) The distance between the SPB and MTs. Models from tomograms of seven *hrs1^+^* cells and six *hrs1* deletion cells were used for the measurements. The distance from the surface of the MT in the model to the surface of the SPB was measured for the closest point of each MT. In total, 147 MTs for the *hrs1^+^* and 57 MTs for the *hrs1* deletion cells were scored and percent distribution against the total MTs was plotted. (D) The distance from the SPB and MT ends. Same tomograms as in (C) were used for the measurements. The distance between the MT ends in the model to the surface of the SPB was measured for the closest end of each MT. In total, 149 MT ends for the *hrs1^+^* and 49 MT ends for the *hrs1* deletion cells were scored and percent distribution against the total MTs was plotted. Seven MTs in the *hrs1* deletion cells were passing through the complete model and had no ends to be measured. Those MTs are included in the over 420 nm category. (E) Distribution of vectorial angles of MTs against a vector line along the long axis of the SPB. Angles are presented in 0°–90° range by applying operations as described in Supplemental Information. In total, 147 MTs for the *hrs1^+^* and 57 MTs for the *hrs1* deletion cells were analyzed and percent distribution against the total MTs was plotted.

To explore how the rMTs are organized, we performed electron microscopy (EM) tomography using synchronized cultures. The conventional synchronization method employs *h^−^/h^−^ pat1^ts^/pat1^ts^* diploid cells that exhibit incomplete centromere detachment from the SPB during meiotic prophase and equational chromosome segregation at MI [26–28]. Because mating factor signaling restores normality to these cells [26–28], we employed *h^−^/h^−^ pat1^ts^/pat1^ts^* diploid cells carrying one copy of *mat-Pc* mating-type cassette and refined the culture conditions (see Supplemental Information) to achieve highly synchronous (80%) MI nuclear division followed by an equally highly synchronous MII (Figure 1A). Cells were high-pressure frozen 80 min after induction of meiosis, when vigorous horsetail nuclear movement is underway, and EM tomograms were obtained from the area of the SPB that can be identified as an electron dense structure that assembles at the nuclear envelope (Movie S1). As expected from the previous studies, the rMT structure was found emanating from the area close to the SPB (Figure 1B; Movie S3). However, strikingly, 90% of the MTs were found to be at least 30 nm away from the SPB (Figure 1C), with over 70% of the MT ends in the cytoplasmic area 30–180 nm away from the SPB (Figure 1D). This is in significant contrast to the previously observed interphase SPB where the associated MTs are located in very close proximity to the SPB [29, 30]. Detailed examination of the morphology of these MT ends revealed that most are closed or “capped,” a characteristic feature of MT minus ends [30–32] (Figure 1B). We refer to this area of high MTOC activity as the rMT organizing center (rMTOC). The rMTOC is reminiscent of the PCM of the mammalian centrosome because most of the minus ends of MTs emanate not directly from the core structure of the SPB, but from the rMTOC, as is the case for the centrioles and the PCM. From seven SPBs analyzed, 21.3 ± 4.8 MTs were associated with each SPB and the varying vector directions of these MTs reflect the “radial” array organization (Figure 1E).

To investigate the role of Hrs1 in organizing the rMTOC, we performed EM tomography on SPBs from *hrs1* deletion (*hrs1Δ*) meiotic prophase cells (Movies S2 and S4). In these cells, the number of MTs associated with each SPB dropped substantially to 9.8 ± 3.5; moreover, ≥40% of MTs were now found within 30 nm of the SPB (Figure 1C), suggesting a severe alteration of rMTOC structure. The number of microtubules at each SPB and the short distance between microtubules and the SPB is comparable to the interphase MT-SPB arrangement [29, 30]. Vector directions of these MTs showed little variation (Figure 1E) demonstrating the loss of the radial MT arrangement. In addition to MTs whose ends were found within 30 nm of the SPB, some MT ends localized more than 200 nm away in *hrs1Δ* cells (Figure 1D), indicating that there is no strong MTOC activity associated with the SPB. Overall, the arrangement of the MTs and the SPB in the absence of Hrs1 more closely resembles that of cells in mitotic interphase [30]. Similarly, at the later time point (120 min), our preliminary observation of the thin sections showed that the MTs are at a very close proximity to the SPB in the *hrs1Δ* samples (For example, see Figure S1C) indicating that the rMTOC structure is completely missing, rather than delayed, in the absence of Hrs1. The result is consistent with our observation by fluorescent microscopy that rMT formation is abolished throughout the meiotic prophase in the *hrs1Δ* cells [20].

### Hrs1 Is Enriched at the rMTOC

**Figure 2:**
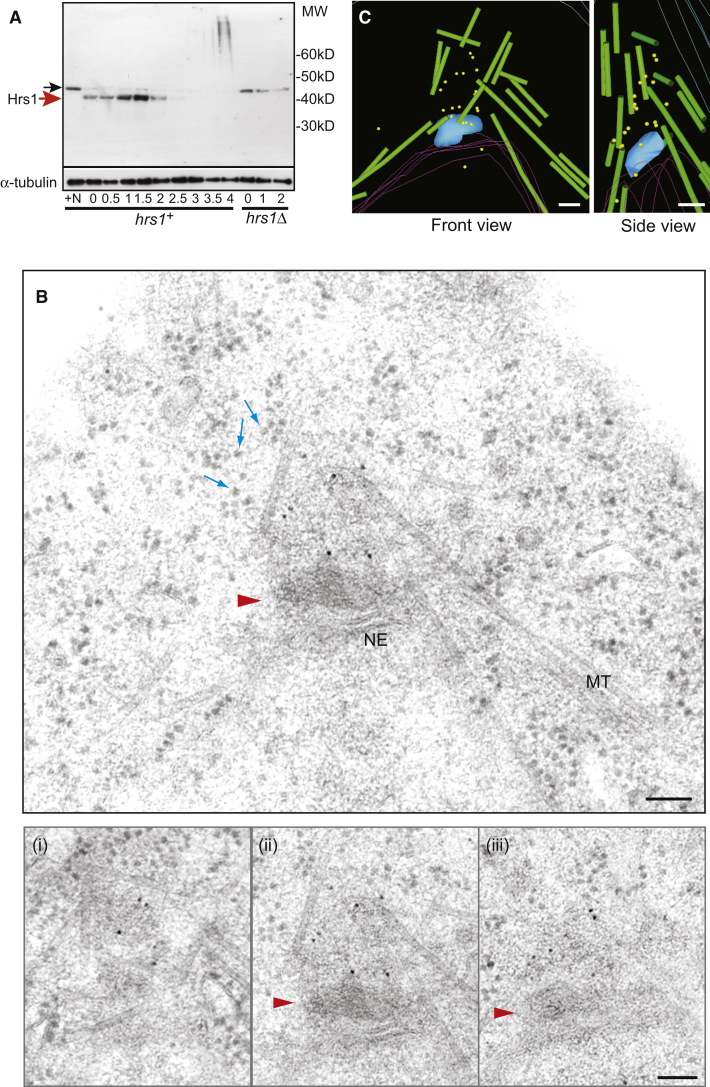
Hrs1 Is Found Enriched at the rMTOC Site (A) Validation of Hrs1 antisera. Whole-cell lysates of the synchronous *hrs1^+^* cells undergoing meiosis as presented in Figure 1A, and *Δhrs1* cells were subjected to SDS-PAGE and western blotting with the Hrs1 antisera and anti-α-tubulin antibody TAT1. “+N” indicates vegetatively growing cells in YE media with a rich nitrogen source. Numbers indicate hr after induction of meiosis by temperature shift. A doublet signal specific to the *hrs1^+^* cells (red arrow) and a nonspecific band (black arrow) are indicated. (B) Hrs1 antisera were used for immunogold labeling/transmission electron microscope (TEM). Diploid *h^−^ pat1.114 mat-Pc* cells harboring WT *hrs1* allele were induced for synchronous meiosis for 80 min (Figure S1B, left panel). Three serial sections (i)–(iii) spanning a SPB and a larger view of the section (ii) are presented. Hrs1 antisera were detected by 10 nm colloidal gold-conjugated protein A. The red arrowhead indicates the SPB. Blue arrows indicate a few ribosomes, which appear as small round electron-dense structures of about 15 nm diameter. MT, microtubules; NE, nuclear envelope. Scale bar represents 100 nm. (C) 3D model of immunogold localization of Hrs1 in five serial sections through a single SPB presented in Figure 2B. Yellow, Hrs1 labels; green, MTs; blue, SPB. Outlines of NE and plasma membrane are indicated by pink and light blue lines, respectively. Scale bar represents 100 nm.

Our identification of the rMTOC, along with the observation that Hrs1 promotes its formation indicated that Hrs1 may be a prominent component of the rMTOC. Therefore, we examined the precise localization of Hrs1 by immuno-EM. We generated anti-Hrs1 antisera and characterized it by western blot on whole extracts of cells undergoing synchronous meiosis (Figure 1A; Figure 2A). The Hrs1 anti-sera strongly recognize a doublet band at approximately 40 kDa, the predicted molecular weight of Hrs1; this band is absent in *hrs1Δ* extracts (Figure 2A, red arrow). As previously reported [19, 20], the expression of Hrs1 is confined exclusively to meiotic prophase, disappearing approximately 2 hr after induction of meiosis, when MI commences (Figure 2A).

**Figure 3:**
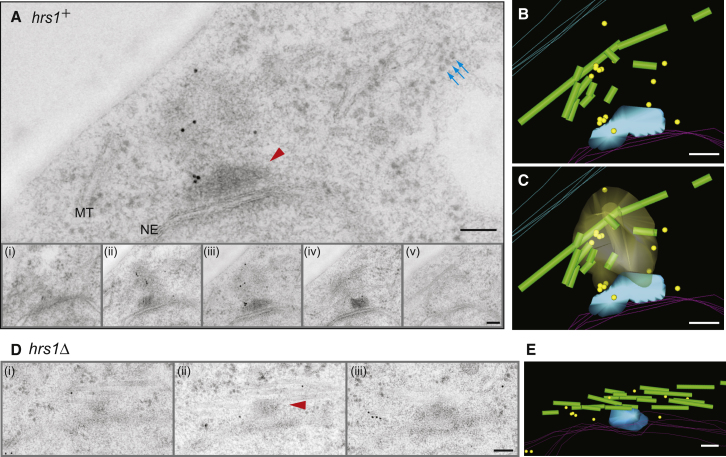
γ-Tubulin Is Found Enriched at the rMTOC Site but Is Missing from the Nuclear Plasmic Side of the SPB (A) Anti-γ-tubulin monoclonal antibody GTU88 was used for EM with immunogold labeling. Diploid *h^−^ pat1.114 mat-Pc* cells harboring WT *hrs1* allele were processed for immunogold labeling as in Figure 2. Five serial sections (i)–(v) spanning a SPB and a larger view of the section (iii) are presented. Anti-γ-tubulin monoclonal antibody was detected by 10 nm colloidal gold-conjugated protein A. The red arrow indicates the SPB. Blue arrows indicate a few ribosomes, which are captured as small round electron-dense structure of about 15 nm diameter. MT, microtubules; NE, nuclear envelope. Scale bar represents 100 nm. (B) 3D model of immunogold localization of γ-tubulin in the five serial sections presented in Figure 3A. Yellow, γ-tubulin labels; green, MTs; blue, SPB. Outlines of the NE and plasma membrane are indicated by pink and light blue lines, respectively. Scale bar represents 100 nm. (C) 3D model of immunogold localization of γ-tubulin as in Figure 3B with an indication of ribosome-free amorphous structure highlighted by light beige. (D) The localization of γ-tubulin was analyzed in the diploid *h^−^ pat1.114 mat-Pc* cells harboring *hrs1* deletion allele. Cells were processed as in the Figure 3A and three serial sections (i)–(iii) spanning a SPB are presented. The red arrow indicates the SPB. Scale bar represents 100 nm. (E) 3D model of immunogold localization of γ-tubulin in six serial sections including the sections (i)–(iii) presented in the Figure 3D. Yellow, γ-tubulin labels; green, MTs; blue, SPB. Outlines of the NE are indicated by pink lines. Scale bar represents 100 nm.

Using the anti-Hrs1 sera, thin sections of cells undergoing meiotic prophase (time 80 min, Figure S1B, left panel) were labeled and detected by 10 nm gold conjugated protein A. Distinct Hrs1 signals were observed at the cytoplasmic side of the SPB corresponding to the rMTOC site (Figures 2B and 2C). The label was confined to an area just outside the SPB and stretched out to a distance of 150–350 nm from the SPB (measured on 20 serial sectioned SPBs). Some sections clearly showed the rMTOC site to be slightly electron dense and ribosome free (see Figure 3A for a clearer example), strongly indicating that some structure occupies this area. No label was found on SPBs in sections from the equivalent *hrs1Δ* strain (Figure S1C). Also there was no interruption in the scattered ribosomes near the SPB in the *hrs1Δ* cells. These results indicate that Hrs1 scaffolds an amorphous structure of the rMTOC and support the notion that Hrs1 bridges the SPB and the minus ends of cytoplasmic MTs.

### γ-Tubulin Is Enriched at the rMTOC

As fission yeast γ-tubulin Gtb1 plays an essential role in MTOC activities during the vegetative cell cycle [33–35], we examined its localization in the rMTOC structure using an anti-γ-tubulin mouse monoclonal antibody (GTU-88) (Figure S2A). First, GTU-88 was validated for EM studies using vegetatively growing interphase cells where extensive EM analyses were performed [36]. As previously observed with anti Gtb1 polyclonal antibodies [36], the majority of GTU-88 signal localizes to the nucleoplasmic side of the interphase SPB (Figure S2B). When GTU-88 was applied to the serial sections of cells 80 min after meiotic induction, γ-tubulin label was found at the rMTOC site, which appears slightly electron dense as mentioned above (Figures 3A–3C). In *hrs1Δ* cells, the γ-tubulin signal was dispersed to a wider area surrounding the SPB (Figures 3D and 3E). Interestingly, regardless of the *hrs1* status, the nucleoplasmic side of the SPB is free of γ-tubulin labeling during meiotic prophase, in striking contrast to its localization in interphase vegetative cells.

### Proteasome-Dependent Disappearance of Hrs1 at the Onset of MI

**Figure 4:**
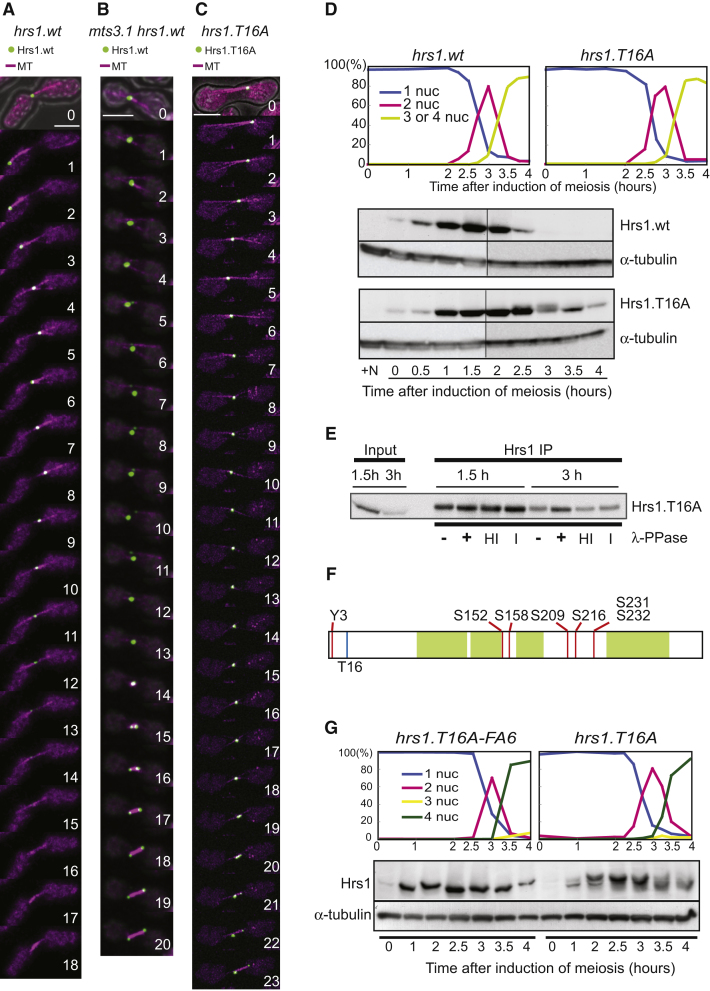
Hrs1 Is Subject to Both Proteasome-Dependent Degradation and Phosphorylation at the Onset of MI Spindle Formation (A) Timing of Hrs1 disappearance was examined using a strain expressing endogenously tagged, functional Hrs1-2×FLAG-GFP and mCherry-tagged α-tubulin Atb2 [60]. Cells of *h^90^ hrs1-2×FLAG-GFP mCherry-atb2* were induced for meiotic differentiation at 30°C and GFP and mCherry signals recorded every min. Each time point comprises 13 serial images of 0.6 μm interval along the z axis taken to span the full thickness of the cell. Deconvolved and Z projected images are presented. (B) Cells of *h^90^ mts3^ts^ hrs1-2×FLAG-GFP mCherry-atb2* were induced for meiotic differentiation at the semipermissive temperature (30°C) and GFP and mCherry signals recorded as in (A). (C) Cells of *h^90^ hrs1.T16A-2×FLAG-GFP mCherry-atb2* were induced for meiotic differentiation and GFP and mCherry signals recorded as in (A). (D) Diploid *pat1.114 mat-Pc* cells harboring either *hrs1.WT-2×FLAG-GFP* or *hrs1.T16A-2×FLAG-GFP* were synchronized at G1 by nitrogen starvation and induced for meiosis by temperature shift. Synchronicity was monitored by counting the number of nuclei (top two panels). Whole-cell lysates were prepared at the indicated time points after induction of meiosis (lower two panels). “+N” indicates vegetatively growing cells. Western blotting was performed with an anti-GFP antibody. α-tubulin was used as a loading control. Note that samples of +N, time 0, 0.5, 1, 1.5, and 2 hr of both Hrs1.WT and Hrs1.T16A were originally loaded on the same gel and samples of time1.5, 2, 2.5, 3, 3.5, and 4 hr of both Hrs1.WT and Hrs1.T16A were loaded on the other gel. These two blots were aligned along the time course, to appreciate their expression profiles throughout meiotic differentiation, referring to the signal intensities of samples 1.5 and 2 hr both of which were included in both membranes. (E) Slow-migrating species of Hrs1 is sensitive to phosphatase treatment. Hrs1 was immunoprecipitated from the *hrs1.T16A-2×FLAG-GFP* cells presented in the Figure 4D. The immunocomplexes from cells at 1.5 hr and 3 hr were treated with λ-PPase and analyzed on a 10% polyacrylamide gel. Smear fuzzy signals observed in the 3 hr sample were packed down after the phosphatase treatment. HI, treatment with heat-inactivated λ-PPase; I, λ-PPase treatment in the presence of inhibitors. (F) Summary of the phosphorylated Hrs1 residues found in this study. Phosphorylated Y3, S152, S158, S209, S216, S231, and S232 were found (indicated by red lines). Location of T16, where an alanine substitution mutation (T16A) was introduced to improve Hrs1 protein stability which was essential for preparation of Hrs1 for LC-MS/MS analysis, is indicated by a blue line. Coiled-coil regions predicted by COILS [45] are indicated by green boxes. (G) The identified seven residues represent major phosphorylation sites. Diploid *pat1.114 mat-Pc* cells harboring either *hrs1.T16A-FA6 −2×FLAG-GFP* (nonphosphorylatable and stabilized mutation) or *hrs1.T16A-2×FLAG-GFP* (stabilized mutation) were synchronized at G1 by nitrogen starvation and meiosis was induced by a temperature shift. Synchronicity was monitored by counting the number of nuclei (top two panels). At the indicated times on the bottom panel, whole-cell lysates were prepared and run on a SDS-PAGE gel supplemented with 40 μM Phostag. Western blotting was performed with an anti-GFP antibody. α-tubulin was used as a loading control (lower panel).

Having observed the rMTOC to be a prominent and Hrs1-dependent structure, we speculated that, for a smooth transition into the next differentiation stage, MI, Hrs1 may need to be removed to dismount the rMTOC. To explore this possibility, we examined the timing of Hrs1 disappearance (Figure 4A). During the period of horsetail nuclear movement, Hrs1-2×FLAG-GFP clearly localized to the SPB, as previously observed [19, 20]. However, as nuclear movement came to an end, both cytoplasmic MTs and Hrs1-2×FLAG-GFP signals became weaker. Within a few minutes of the disappearance of the Hrs1-2×FLAG-GFP signal, formation of the MI spindle commences (Figure 4A, time 14 to 16; Movie S5).

Although the expression of *hrs1* messenger RNA (mRNA) sharply drops at the time of MI entry [37], we suspected that the abrupt disappearance of Hrs1 protein might involve additional posttranslational regulation, such as proteasome-dependent degradation. Indeed, the Hrs1-2×FLAG-GFP signal persists on the SPB(s) throughout MI when introduced into a temperature-sensitive proteasome mutant, *mts3.1* [38], confirming that Hrs1 is a target of proteasome-dependent degradation (Figure 4B). When His6 tagged ubiquitin was expressed either in wild-type (WT) or *mts3.1* mutant strains, followed by Ni purification and Hrs1 western blot, ubiquitylated Hrs1 was readily detected in the *mts3.1* mutant cells (Figure S3A). The result further supports the observation that Hrs1 is subject to proteasome-dependent degradation.

Deletion and mutational analyses of Hrs1 identified threonine 16 as a critical residue for the Hrs1 stability at the MI onset (Figure S3B). Mutant Hrs1 harboring an alanine substitution at this site (Hrs1.T16A) persisted at the SPB through MI (Figure 4C). The Hrs1.T16A was found fully capable of assembling the rMTs and of inducing horsetail nuclear movement (data not shown). Interestingly, the intensity of the Hrs1.T16A-2×FLAG-GFP signal on the separating spindle poles tended to be uneven, implying that Hrs1 may preferentially associate with either the older or newer SPB (Figure 4C). The mRNA expression profile of *hrs1.T16A-2×FLAG-GFP* was comparable to that of *hrs1.WT-2×FLAG-GFP* (Figure S3C and S3D), indicating that the T16A mutation affects protein stability rather than transcriptional regulation. The protein level of Hrs1.T16A-2×FLAG-GFP during meiotic prophase is comparable to the one of Hrs1.WT-2×FLAG-GFP (Figure 4D, time 0–1.5 hr). However, whereas Hrs1.WT-2×FLAG-GFP starts to disappear at the onset of MI, Hrs1.T16A-2×FLAG-GFP remains present throughout meiotic differentiation (Figure 4D, time 2–4 hr).

### Phosphorylation of Hrs1 Regulates Its MTOC Function

Intriguingly, western blot analysis of extracts from *hrs1.T16A* cells at MI onset (Figure 4D, time 2.5 hr and 3 hr) showed a smeared band of stabilized Hrs1.T16A, which was resolved by phosphatase treatment (Figure 4E). To identify the phosphorylated residues, we purified the Hrs1.T16A-2×FLAG-GFP from cells undergoing the transition from meiotic prophase to MI (Figure S4A). The purified Hrs1.T16A-2×FLAG-GFP was then analyzed by liquid chromatography and tandem mass spectrometry (LC-MS/MS) to reveal seven phosphorylated residues (Y3, S152, S158, S209, S216, S231, and S232; Figure 4F; Figures S4B–S4H).

To explore the functional relevance of Hrs1 phosphorylation, we generated a mutant allele, *hrs1.T16A-FA6*, which encodes a nonphosphorylatable and stabilized Hrs1 protein, by mutating all identified phosphorylation sites with either phenylalanine or alanine (Y3F-S152A-S158A-S209A-S216A-S231A-S232A) in addition to the T16A stabilizing mutation. The modification status of Hrs1.T16A-FA6 was analyzed by SDS-PAGE supplemented with Phostag [39] to help resolve phosphorylated species. Whereas the Hrs1.T16A showed several differently migrating Hrs1 species, Hrs1.T16A-FA6 showed one major band, indicating that the majority of posttranslational modifications were abolished in the Hrs1.T16A-FA6 mutant (Figure 4G). We also generated a phosphomimetic version of Hrs1, Hrs1.D7, in which Y3, S152, S158, S209, S216, S231, and S232 are all mutated to aspartic acid.

**Figure 5:**
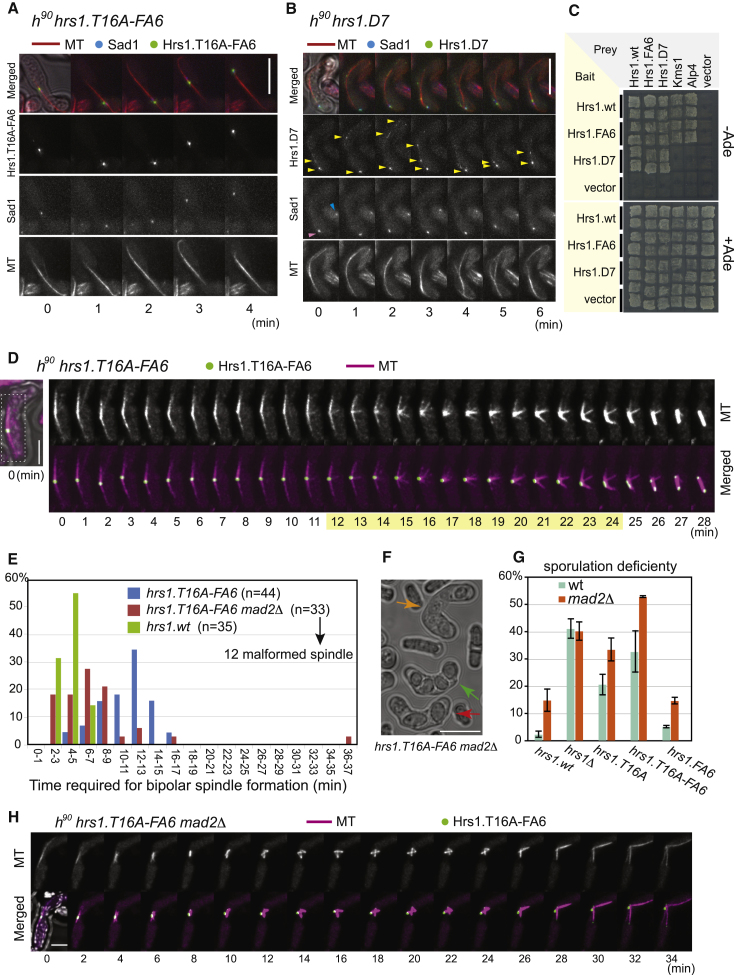
Phosphorylation of Hrs1 Downregulates Hrs1 Function to Facilitate Meiosis I Bipolar Spindle Formation (A) Vigorous horsetail movement and rMT formation observed in the presence of Hrs1.T16A-FA6, a stabilized and nonphosphorylatable mutant. Cells of *h^90^ hrs1.T16A-FA6-2×FLAG-GFP mCherry-atb2 sad1-CFP* were induced for meiotic differentiation at 30°C and GFP, CFP, and mCherry signals recorded as in Figure 4A. Sad1-CFP was used as an SPB marker. Numbers indicate the minutes after the start of the film. Scale bar represents 10 μm. (B) An example of disorganized rMT in the presence of the *hrs1.D7* allele. Cells of *h^90^ hrs1.D7-2×FLAG-GFP mCherry-atb2 sad1-CFP* were induced for meiotic differentiation at 30°C and GFP, CFP and mCherry signals recorded as in Figure 4A. Sad1-CFP was used as an SPB marker. Scattered Hrs1.D7 foci are indicated by yellow arrowheads. Sad1 signal in the zygote undergoing meiotic prophse is indicated by a red arrowhead and Sad1 signal in the neighboring cell is indicated by a blue arrowhead. Numbers indicate the min after the start of filming. Scale bar represents 10 μm. (C) Hrs1.D7 failed to interact with Kms1 or Alp4 in yeast two hybrid assays. Two independent colonies of the budding yeast host strain AH109 carrying the bait and prey plasmids were incubated either in the absence (upper panel) or presence (lower panel) of adenine. (D) A typical example of a transient monopolar spindle generated in the presence of the *hrs1.T16A-FA6* (stabilized and nonphosphorylatable) allele in otherwise WT cells. MTs are tagged with mCherry (magenta) and Hrs1.T16A-FA6 is tagged with 2×FLAG-GFP (green). Images were recorded as in Figure 4A. Numbers indicate the minutes after the start of filming. The time spent to establishing a bipolar spindle is highlighted with light yellow. Only the blown up area, indicated by a dotted line in the merged image at time 0 (far left panel), is presented. Scale bar represents 5 μm. (E) Duration between disappearance of the cytoplasmic MTs and start of the bipolar spindle formation. WT or *Δmad2* cells harboring *hrs1.T16A-FA6*-2×FLAG-GFP and mCherry-*atb2* were induced for meiosis and live cell imaging was performed. The distribution of populations of zygotes that spent the corresponding time duration establishing bipolar spindles is presented. As a control, WT cells harboring *hrs1.WT-2×FLAG-GFP* and *mCherry-atb2* were used. (F) Examples of aberrant spore formation observed in *hrs1.T16A-FA6 Δmad2* cells. Normal spore formation gives four clear spores (indicated by green arrow); however, zygotes with ill-defined spores (orange arrow) or with wrong number of spores (red arrow) were observed. The latter two were counted for zygotes with aberrant spore formation in Figure 5G. Scale bar represents 10 μm. (G) Sporulation deficiency observed in WT and *Δmad2* cells harboring various *hrs1* mutant alleles. Percentages of zygotes harboring aberrant spores are presented. Cells were induced for sporulation on sporulation agar medium at 25°C. For each genotype, two independently isolated strains of identical genotypes were used and 400–450 zygotes were counted for each case. (H) An example of a malformed spindle in a *Δmad2* cell harboring the *hrs1.T16A-FA6* allele. Homothallic *Δmad2 hr1.T16A-FA6-2×FLAG-GFP mCherry-atb2* cells were induced for meiotic differentiation at 30°C, and GFP and mCherry signals were recorded as in Figure 4A. Numbers indicate the minutes after the start of filming. Scale bar represents 5 μm.

As expected, based on the major phosphorylation events to appear upon MI onset, rMT organization appeared normal in cells carrying the nonphosphorylatable Hrs1.T16A-FA6-2×FLAG-GFP (Figure 5A). However, the phosphomimetic Hrs1.D7-2×FLAG-GFP was often detected as multiple foci of weaker signal intensities, whereas the rMT appeared disorganized (Figure 5B). These observations support the idea that Hrs1 phosphorylation promotes rMT dissolution and that such phosphorylation must therefore be restricted to postmeiotic prophase cells to allow rMTOC to function during meiotic prophase.

To address the basis for Hrs1 phosphorylation-induced transitions in rMT organization, we examined several relevant Hrs1 protein interactions by yeast two hybrid analysis. Both mutants retain their abilities to oligomerize with themselves, suggesting that they were properly expressed and folded (Figure 5C). Strikingly, whereas the nonphosphorylatable mutant Hrs1.FA6 behaves like WT Hrs1, by interacting with both Kms1 and Alp4 (Figure 5C), Hrs1.D7 fails to interact with Kms1 and Alp4. However, Hrs1.D7 retains the ability to interact with the second KASH-domain protein, Kms2 (data not shown). Hence, the residual rMT formation and affinity to the SPB seen in *hrs1.D7* cells (Figure 5B) may be afforded by Hrs1-Kms2 interactions to compensate for compromised interactions with Kms1 and Alp4.

### Stabilized Nonphosphorylatable Hrs1 Interferes with Meiotic Bipolar Spindle Formation

Although Hrs1.T16A-FA6 is capable of acting as an rMT organizer during meiotic prophase, its persistence at the transition between meiotic prophase and MI causes spindle defects. In *hrs1.T16A-FA6* cells, a monopolar spindle forms transiently before establishment of a bipolar spindle (Figure 5D, time 12 to 24 min). Consequently, whereas bipolar spindle formation occurs immediately after cytoplasmic MTs disappear in WT cells (Figures 4A and 5E), a delay of 10 to 15 min is seen in the presence of Hrs1.T16A-FA6 (Figure 5E). This delay was not obvious in the *pat1^ts^ mat-Pc* diploid synchronous system, presumably because at its higher culturing temperature (34°C), the delay became shorter or abrogated.

Delayed mitosis in response to compromised chromosome-spindle interactions depends on activation of the spindle assembly checkpoint (SAC) where Mad2 plays the central role [40]. Strikingly, deletion of *mad2* abolished the delay to bipolar spindle formation by Hrs1.T16A-FA6 (Figure 5E). Moreover, about one third (12 out of 33 zygotes observed) of *hrs1.T16A-FA6 mad2Δ* zygotes generated aberrant spindles (Figure 5H). This leads to exacerbated sporulation deficiency over 50% (Figures 5F and 5G). A *hrs1.FA6* strain, which harbors all the nonphosphorylatable substitutions but lacks the T16A stabilizing mutation, does not show a substantial sporulation deficiency in the *mad2Δ* background, indicating that Hrs1 interferes with MI spindle formation only when it escapes both inhibitory mechanisms, namely proteasome-dependent degradation and phosphorylation.

### SPB Duplication Occurs in Early Meiotic Prophase

**Figure 6:**
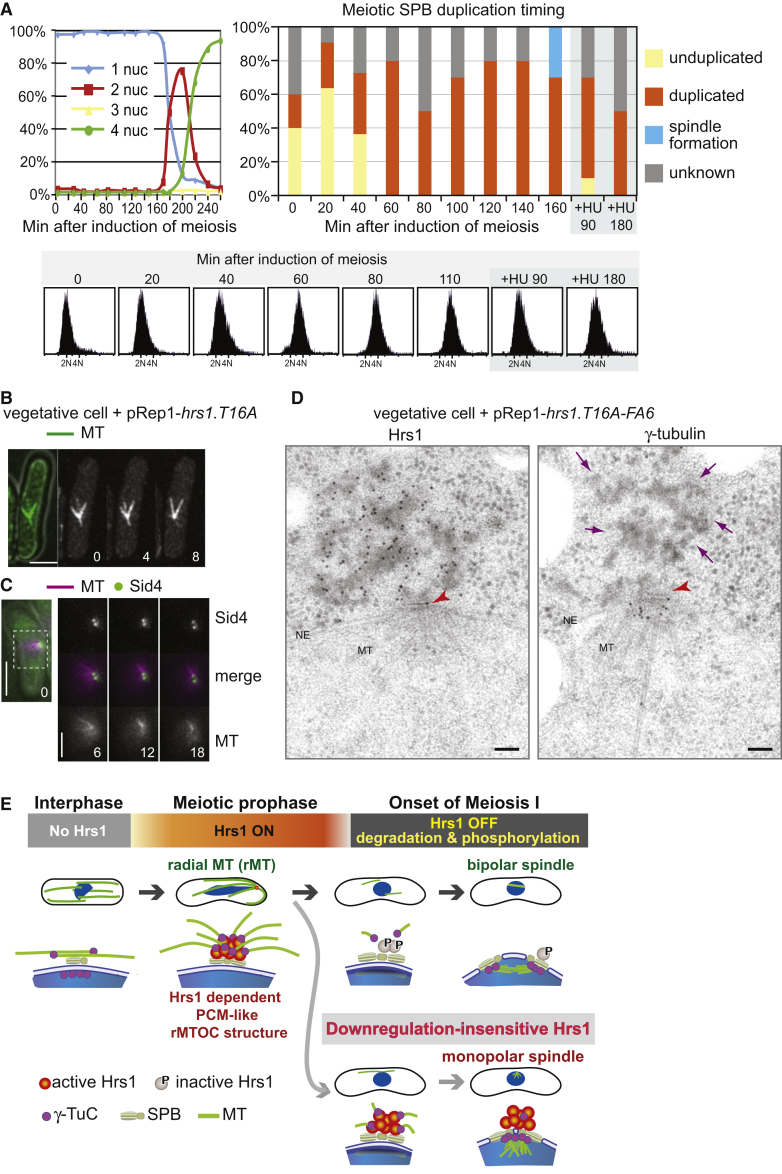
Persisting Hrs1 Disturbs Bipolar Spindle Formation Possibly by Tethering the Separating SPBs (A) Meiotic SPB duplication occurs early in meiotic prophase. Highly synchronous meiotic culture of a diploid *h^−^ pat1.114 mat-Pc* strain was used for the SPB duplication assay. Upper left panel shows that synchronicity was monitored as in Figure 1A. Upper right panel shows that cells at the indicated time points were high-pressure frozen and processed for TEM analysis. Serial sections spanning a SPB were analyzed for 10–11 cells at each time point. When two lamellar bodies were recognizable, the SPB was counted as “duplicated”; if only one lamellar body was present, it was referred as an “unduplicated” SPB. In total, ten SPBs were analyzed at each time point except for 20 min and 40 min where 11 SPBs were analyzed. “+HU” indicates cells treated with 12 mM hydroxyurea. Lower panel presents the corresponding DNA content profile by FACS analysis. (B) MT arrangements are altered by ectopic expression of *hrs1.T16A* that encodes the stabilized Hrs1.T16A mutant protein from the strong inducible *nmt1* promoter [44] for 22 hr. A representative image of a monopolar spindle in a WT cell ectopically expressing the Hrs1.T16A mutant protein. GFP-α-tubulin signal was recorded at 4 min intervals. Scale bar represents 5 μm. (C) Two Sid4-GFP signals were found at the pole of a monopolar spindle induced by ectopic expression of Hrs1.T16A. The Hrs1.T16A was overexpressed in WT cells harboring *sid4-GFP* and *mCherry-atb2.* Sid4-GFP and mCherry-Atb2 (mCherry-MT) signals were recorded at 6 min intervals. Only the blown-up area, indicated by a dotted square in the merged image at time 0 (far left panel), is presented. Scale bar represents 5 μm. (D) Hrs1 antisera and anti-γ-tubulin monoclonal antibody were used for EM with immunogold labeling/TEM. WT cells expressing Hrs1.T16A-FA6 were high-pressure frozen and processed for immunogold labeling. Hrs1 antisera and anti-γ-tubulin monoclonal antibody were detected by 10 nm colloidal gold-conjugated protein A. The red arrowhead indicates the SPB. Purple arrows point out the amorphous electron-dense aggregation formed at the cytoplasmic side of the SPB. MT, microtubules; NE, nuclear envelope. Scale bar represents 100 nm. (E) A proposed model. During meiotic prophase, Hrs1 plays an essential role in rMT formation by generating the rMTOC, an amorphous structure at a cytoplasmic site associated with the SPB. γ-tubulin is also enriched in the rMTOC. At the transition to MI, Hrs1 is removed by proteasome-dependent degradation. Furthermore, any remaining Hrs1 molecules are inactivated by phosphorylation to ensure bipolar spindle formation. The downregulation-insensitive Hrs1 disturbs bipolar spindle formation by interfering with separation of the duplicated SPBs. This may be achieved by Hrs1, or the rMTOC, acting as a “glue” to hold duplicated SPBs together. Alternatively, stabilized Hrs1 may titrate out the γ-TuC necessary for spindle formation. Although these two possibilities are not mutually exclusive, we prefer the former hypothesis because ectopically expressed Hrs1 in vegetative cells aggregated at the cytoplasmic side of the SPB without apparently affecting γ-tubulin accumulation at the nucleoplasmic side of the SPB (Figure 6D).

The impairment in meiotic SPB separation and bipolar spindle formation conferred by Hrs1.T16A-FA6 suggests that persisting Hrs1 may hold the duplicated meiotic SPBs together. If this is the case, duplication of the SPB is expected to occur early in meiotic prophase and Hrs1 decorates both the duplicated SPBs rather than a single unduplicated SPB. To confirm this prediction, we examined the timing of SPB duplication during meiotic prophase. Preliminary attempts to use Sid4-GFP signal intensity to monitor the duplication timing was not successful, because we did not detect distinct doubling of the intensity; instead, a marginal increase was observed until the intensity dropped to the half when the duplicated SPBs were separated by the MI spindle (data not shown). Therefore, we examined individual SPBs using transmission EM, analyzing serial sections spanning the entire SPB. Although the nature of the assay did not allow us to handle large number of cells, at least ten SPBs were analyzed for each time point to capture a trend for duplication timing (Figure 6A). Unduplicated SPBs were only found at early time points (0–40 min), and the duplication was completed by 60 min, coinciding with the timing of bulk DNA replication (40–60 min), although completion of DNA replication and SPB duplication are independent as was revealed by hydroxyurea treatment (Figure 6A). As the MI spindle formation started at around 160 min (Figure 6A), our observation suggests that the SPB is in the duplicated status for about 100 min, during which time the rMTOC is expected to decorate the SPB. Also, in EM tomograms of SPBs at 80 min, when the rMTOCs have been well established, two duplicated lamellar bodies [41] (a laminate structure that sits next to the bridge structure of the SPB) were recognized, i.e., the SPB had duplicated (for example, Figure 1C). These results confirm that the rMTOC structure exists on duplicated SPBs and supports the hypothesis that delayed rMTOC dissolution interferes with separation of the duplicated meiotic SPBs.

### Expression of Hrs1 in Mitotic Cells Interferes with Bipolar Spindle Formation

If persisting Hrs1 interferes with separation of the duplicated SPBs at the MI onset, Hrs1 is expected to also affect mitotic spindle formation when ectopically expressed. To test this hypothesis, we exploited vegetatively growing cells as a “test tube” in which Hrs1.WT or Hrs1.T16A was ectopically expressed. Strains harboring GFP-tagged α-tubulin (*GFP-atb2*) [42] were used for live imaging and cells classified into categories based on MT structure (Figure S5A).

Upon expression of Hrs1.WT or Hrs1.T16A, interphase parallel MT bundles were converted into rMT-like structures as previously observed [20] (Figures S5A and S5B). Strikingly, in mitosis, cells with a monopolar spindle (monopolar spindle delay, Figure 6B) or with a short spindle of constant length (mitotic delay) accumulated, especially upon induction of Hrs1.T16A (Figure S5D). Examination of a GFP-tagged SPB component, Sid4 [43], clearly revealed two SPB signals in cells containing monopolar spindles, indicating that the SPBs had duplicated (Figure 6C). Accumulation of these cells is dependent on the SAC as abrogation of SAC by *mad2* deletion enabled cells to progress to anaphase (Figures S5C and S5D) with spindles of aberrant morphology (Figure S5C). This led to severe chromosomal abnormalities (Figures S6A–S6C), leading to a lethal phenotype.

Having observed that the stabilized Hrs1.T16A was more effective than Hrs1.WT in inducing monopolar spindle and lethality in the *mad2Δ* cells, we examined cell viabilities upon expression of various Hrs1 mutants under an intermediate strength *nmt41* promoter [44] in *mad2Δ* cells. Viability then is expected to reflect the ability of each Hrs1 mutant to disturb bipolar spindle formation. Whereas Hrs1.D7 did not affect cell viability, Hrs1.T16A, Hrs1.FA6, and Hrs1.T16A-FA6 resulted in a significant loss of viability (Figure S6D). Importantly, among all the Hrs1 mutants examined, Hrs1.T16A-FA6, the stabilized and nonphosphorylatable mutant, brings about the most severe viability loss confirming that degradation and phosphorylation independently downregulate Hrs1 function.

To further explore how Hrs1 interferes with the bipolar spindle formation, we examined the localization of ectopically expressed Hrs1.T16A-FA6-HA by immuno-EM. Although a few electron-dense cytoplasmic aggregations of Hrs1.T16A-FA6 were found in places other than the SPB, when monopolar spindles were recognized, they were associated with a SPB heavily decorated by the Hrs1.T16A-FA6 from the cytoplasmic side (Figure 6D, left panel). The majority of γ-tubulin in these spindles is found in the nucleoplasm, as in the control (Figure 6D, right panel). This observation supports the hypothesis that the delay to bipolar spindle formation is caused by Hrs1 holding two SPBs together, rather than γ-tubulin being sequestered to the cytoplasmic side.

## Discussion

The dynamic rearrangement of MT architecture that occurs during cell-cycle progression and differentiation are dictated, at least in part, by the MTOC. In this study, we uncovered that a specialized MTOC is transiently generated during meiotic differentiation. During meiotic prophase, a specific structure, which we termed the rMTOC, is generated to direct vigorous chromosome movement. At the onset of MI spindle formation, however, rMTOC is completely inactivated to facilitate bipolar spindle formation (Figure 6E).

It was previously assumed that the rMTs directly emanate from the core SPB. However EM tomography and immunogold labeling approaches demonstrate that a novel structure, the rMTOC, which resides in the vicinity of the SPB, mediates rMT assembly. The rMTOC was observed as a faintly electron-dense amorphous structure that excludes ribosomes and associates with γ-tubulin. These features are highly reminiscent of the higher eukaryotic PCM, an amorphous structure enriched in γ-tubulin [25]. In the *hrs1* deletion mutant, the rMTOC is missing and γ-tubulin accumulation and rMT formation are abrogated. Hence, Hrs1 plays the primary role in generating the rMTOC, which in turn arranges the rMTs that direct horsetail nuclear movement. Hrs1 harbors, as is often the case for the SPB or centrosome components, coiled-coil motifs [45]. Together with our observations that Hrs1 interacts with itself and Hrs1 overexpression produces electron-dense aggregates, we propose that Hrs1 serves as a structural scaffold for the rMTOC.

Persisting rMTOC is capable of interfering with MI bipolar spindle formation. We believe that this is why Hrs1, the key component of rMTOC, is subject to strict downregulation at the transition from meiotic prophase to MI. This behavior is evocative of the mammalian centrosomal protein Nlp, an interphase MT minus-end anchoring protein that requires prompt removal before mitotic spindle formation, although a direct structural contribution of Nlp to the centrosome is unknown [46]. Downregulation of Hrs1 at MI onset is mediated by a combination of two mechanisms: proteasome-dependent degradation and phosphorylation. We believe that these two mechanisms work independently because we saw only a marginal delay in spindle formation either in the *mts3.1* proteasome mutant, *hrs1.T14A* stabilized mutant, or in the *hrs1.FA6* nonphosphorylatable mutant, presumably because, when one of either regulation (degradation or phosphorylation) is missing, the remaining regulation still turns off the Hrs1 function. In addition, it is entirely feasible that priming of degradation is also regulated by phosphorylation because degradation is blocked by mutation of T16.

Identification of the E3 ubiquitin ligase(s) and kinase(s) responsible for Hrs1 modification will be crucial to reveal the precise regulatory mechanisms. Preliminary observations that the stability of Hrs1 is not altered by a *skp1* temperature-sensitive mutation [47] or *slp1 cut23* shut-off [48] suggest that neither the SCF nor APC/C plays a major role in Hrs1 regulation, although these conditional mutations may retain basal level of E3 ligase activities. Because Cdc2 also plays a pivotal role at the onset of MI, we combined the *hrs1.T16A* stabilized mutation with *tws1*, a meiosis-specific mutant allele of *cdc2* [49, 50] but did not observe a significant decrease in sporulation efficiency. However, because Cdc2 activity is not completely lost in the *tws1* mutant, further study is required to rule out a role for Cdc2.

The fine-tuning functions of Hrs1 phosphorylation are potentially complex due to Hrs1 containing at least seven residues that are phosphorylated. Each of these may be differentially regulated with some residues being phosphorylated earlier and playing different roles than others. Although the Hrs1.D7 mutant, where all the phosphorylation sites are mutated to phosphomimetic residues, shows overall decreased MTOC activity, some of the phosphorylation may act to facilitate Hrs1 function. Indeed, already in early meiotic prophase, Hrs1 is highly likely to be phosphorylated to some extent as Hrs1 appears as a doublet on western blotting (Figure 2A). Further careful analyses employing various *hrs1* phosphorylation mutants are required to identify the precise phosphorylation timings and their roles throughout meiotic progression.

Although we observed a delay in bipolar spindle formation in cells expressing Hrs1 protein that is no longer subject to downregulation, we did not observe a complete block of bipolar spindle formation. It may be that Hrs1 is further downregulated by yet another unidentified mechanism. Another possibility is that the forces promoting SPB separation, generated by the kinesin motor protein Cut7 [51, 52], overcome the adverse effect of sustained Hrs1 expression. Indeed, preliminary observations suggested that *cut7^ts^* cells [51], even at their permissive temperature, are more sensitive to ectopic Hrs1 expression than WT cells.

Through this study, we revealed that the Hrs1 provides the molecular basis to mediate the program of MT rearrangement throughout meiotic differentiation. However, the molecular mechanism, with which the interphase parallel cytoplasmic microtubules are rearranged into the rMTs, still needs to be elucidated. Our preliminary observation suggests that the dynein Dhc1 facilitates the process, although the rMTs are eventually generated in the absence of Dhc1 (K.T., unpublished data). Because weak Hrs1 foci are observed along the MTs [20], minus ends of MTs might be trapped by chance at these Hrs1 foci and then somehow slide toward the rMTOC. Thorough analyses on MT dynamics during early meiotic prophase wait to be conducted.

Intriguingly, Hrs1 shows moderate similarity to the NudE protein family (Figures S6E and S6F), which was originally identified through a genetic screen for mutants deficient in nuclear migration in *Aspergillus nidulans*. These include NudC, NudE, NudF, dynein (NudA), and dynactin (NudM) [53, 54]. NudF is homologous to human LIS1, the protein whose mutation confers lissencephaly syndrome [55]. LIS1 interacts with the two mammalian NudE homologs, Nde1 and Ndel1, and dynein to form a tripartite complex which is involved in the wide-range of the cytoplasmic dynein pathways [56, 57]. Remarkably, LIS1 and Nde1 assist the conversion of the dynein holoenzyme into an effective “force-producing” status [58]. Considering that fission yeast nuclear migration is mediated by the rMTs and dynein-dynactin system, it will be interesting to determine whether Hrs1 is a functional ortholog of NudE and how dynein is involved in rMTOC generation. Furthermore, because a role of Ndel1 in dynein-dependent MT self-organization in the meiotic *Xenopus* egg extracts has been reported [59], it is attractive to examine whether Nde1/Ndel1 contributes structurally to a MTOC in higher eukaryotes.

### Conclusion

In summary, our study reveals that the dynamic MT rearrangement during meiotic differentiation involves transient generation of a specialized MTOC, which is later subjected to a rigorous downregulation to support a smooth transition to the next differentiation stage. Because Hrs1 has likely mammalian orthologs, this paradigm of rigorously controlled, transient, specialized MTOC structures may be conserved across species.

## Experimental Procedures

### Yeast Strains and Media

*Schizosaccharomyces pombe* strains used in this study are listed in Table S1. Preparation of highly synchronous meiotic culture and fission yeast gene manipulation are described in the Supplemental Information.

### Antibody Generation

Rabbit polyclonal Hrs1 antibodies were generated using bacterially expressed full-length Hrs1 tagged with His_6_ at its N terminus (Supplemental Information).

### Western Blotting and Immunoprecipitation

Whole-cell extracts were prepared and analyzed by SDS-PAGE (Supplemental Information). When the phosphorylated species of Hrs1 were analyzed, the acrylamide gel was supplemented with Phostag (Wako Chemicals GmbH) at a final concentration of 40 μM.

### Detection of Ubiquitylation of Hrs1

pRep1-6His-Ubi plasmid kindly provided by Dr. Hiroaki Seino. The 6His-UB complexes were prepared and analyzed as described in the Supplemental Information.

### Fluorescent Microscopy

DAPI staining of nuclei to score their number was performed as previously described [20]. Live imaging of the cells was performed as described in the Supplemental Information.

### Electron Microscopy, Tomography, and Immunolabeling

Cells for EM analysis were done as described in the Supplemental Information. The MT organization at the SPB was evaluated by analyzing the distribution of angles between MTs and the long axis of the SPB as described in the Supplemental Information. Immunogold labeling and SPB duplication assays were performed as described in the Supplemental Information.

### Quantitative PCR

Isolation of total RNA, complementary DNA synthesis, and quantitative PCR analyses were performed as described in the Supplemental Information.

### Liquid Chromatography and Tandem Mass Spectrometry

Cell extract preparation, Gel band destaining and washing, In-Gel tryptic digestion, and nLC-MS/MS Analysis were carried out as described in the Supplemental Information.

## Supplemental Information

Document S1. Figures S1–S6, Table S1, and Supplemental Experimental Procedures

Movie S1. Tomogram of WT Strain, Related to Figure 1 WT cells from 80 min after inducing synchronous meiosis. A movie through the 250 nm thick section, which is the middle section from the three sections used for modeling in Figure 1 and Movie S3, is shown. It harbors the complete SPB. Note the two lamellar structures (a duplicated SPB) and that the microtubules are gathering at the SPB from different directions. Some MTs are longitudinally sectioned and some are cross-sectioned. Scale bar represents 200 nm.

Movie S2. Tomogram of *hrs1* Deletion Strain, Related to Figure 1 Cells of *hrs1* deletion strain from 80 min after inducing synchronous meiosis. A movie through the 250 nm thick section, which is the middle section from the three sections used for modeling in Figure 1 and in Movie S4, is shown. It harbors the complete SPB. Note the two lamellar structures (a duplicated SPB) and that the MTs are parallel and longitudinally sectioned. Scale bar represents 200 nm.

Movie S3. Model of WT Strain, Related to Figure 1 A model from the SPB from a WT cell 80 min after inducing synchronous meiosis was made from three consecutive sections of 250 nm thickness. The MTs (green) are ending in the area above the SPB (light blue) which also have two lamellar structures (black). The nuclear envelope is shown in purple and the plasma membrane in turquoise. The MT ends have been identified and are presented by colors. Capped ends (red), blunt ends (blue), sheet (yellow), and undetermined ends are shown in white. Note that capped ends are more abundant around the SPB.

Movie S4. Model of *hrs1* Deletion Strain, Related to Figure 1 A model from the SPB from a *hrs1* deletion cell 80 min after inducing synchronous meiosis was made from three consecutive sections of 250 nm thickness. The MTs (green) are parallel and pass close to the SPB (light blue), which also have two lamellar structures (black). The nuclear envelope is shown in purple and the plasma membrane in turquoise. The MT ends have been identified and are presented by colors. Capped ends (red), blunt ends (blue), sheet (yellow), and undetermined ends are shown in white.

Movie S5. Hrs1 Disappears Abruptly from the SPB Just before Spindle Formation, Related to Figure 4 Cells of *h^90^ hrs1-2×FLAG-GFP mCherry-atb2* were induced for meiotic differentiation at 30°C and GFP and mCherry signals were recorded every 1 min. Each time point comprises 13 serial images of 0.6 mm interval along the z axis taken to span the full thickness of the cell. Deconvolved and Z-projected images are presented as a movie. Four frames are presented per s. The Hrs1-GFP signal is presented in green and the mCherry-Atb2 is presented in magenta. The zygote at the bottom right was also presented in Figure 4A as image sequences.
