# Supplementary material for: Transient Structure Associated with the Spindle Pole Body Directs Meiotic Microtubule Reorganization in S. pombe
Source: Curr Biol. 2012 Apr 10;22(7):562–74. doi: 10.1016/j.cub.2012.02.042 (PMC3382715; doi:10.1016/j.cub.2012.02.042)
Supplement: Document S1. Figures S1–S6, Table S1, and Supplemental Experimental Procedures [file mmc1.pdf]

Current Biology, Volume *22*

Supplemental Information

Transient Structure Associated with the Spindle Pole Body Directs Meiotic Microtubule Reorganization in *S. pombe*

Charlotta Funaya, Shivanthi Samarasinghe, Sabine Pruggnaller, Midori Ohta, Yvonne Connolly, Jan Müller, Hiroshi Murakami, Agnes Grallert, Masayuki Yamamoto, Duncan Smith, Claude Antony, and Kayoko Tanaka

Supplemental Inventory

1. Supplemental Figures and Tables

Figure S1, related to Figures 1 and 2

Figure S2, related to Figure 3

Figure S3, related to Figure 4

Figure S4, related to Figures 4 and 5

Figure S5, related to Figure 6

Figure S6, related to Figure 6

Table S1

2. Supplemental Experimental Procedures

3. Supplemental References

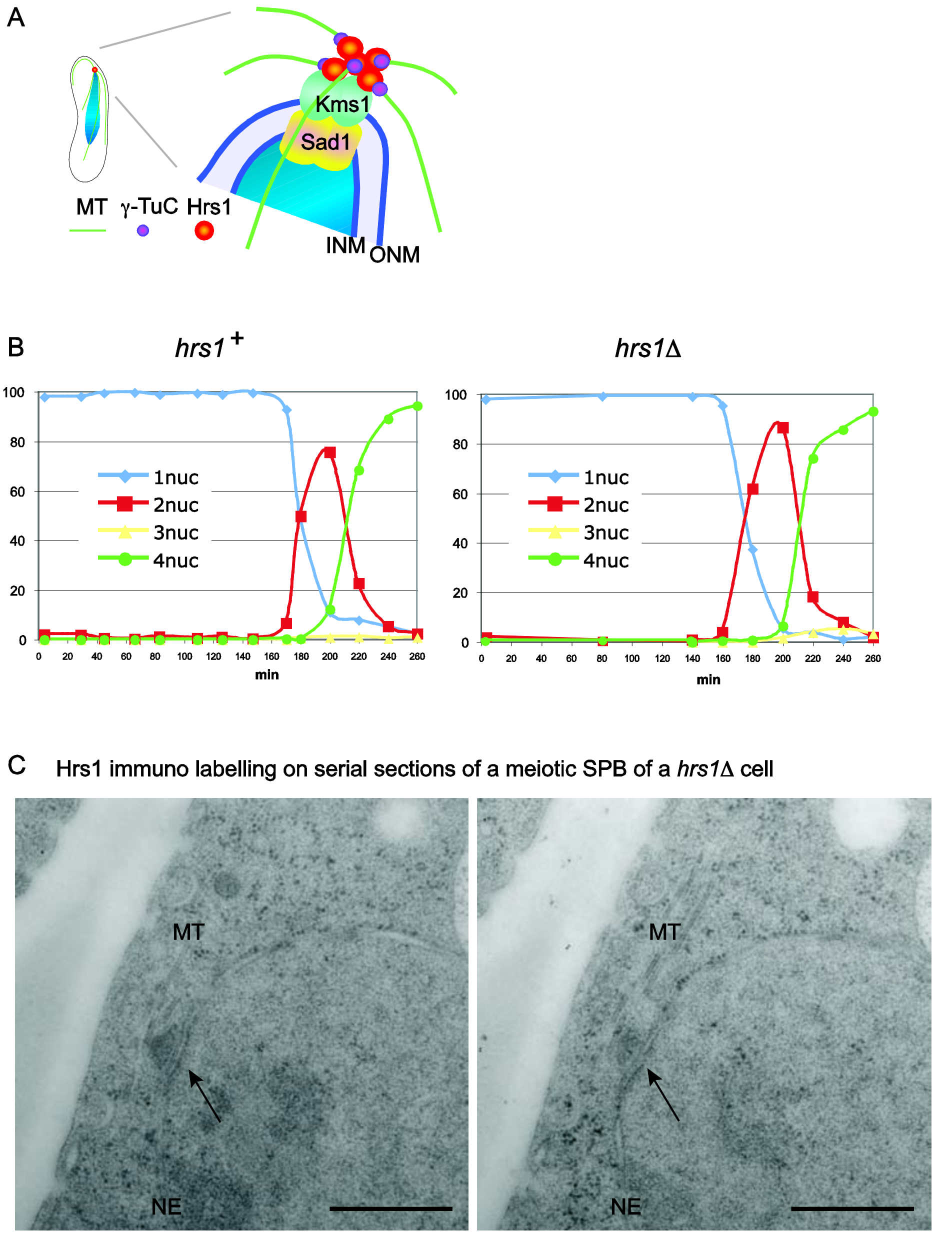

Figure S1. Our Working Hypothesis and Validation of Hrs1 Antisera, Related to Figures 1 and 2

(A) A working model shows Hrs1 oligmers bridging Kms1 and γ-TuC, which decorates the minus ends of MTs, to organize the rMTs. ONM : outer nuclear membrane, INM : inner nuclear membrane.

(B) Synchronicity of meiosis of cell cultures used for EM tomography and immuno EM analyses. KT2486 *hrs1*$^{+}$ cells and KT2919 *hrs1* deletion (*hrs1Δ*) cells were synchronised at G1 by nitrogen starvation before meiosis was induced by a temperature shift and nuclear number was counted. Time (min) after induction of meiosis is indicated.

(C) The *hrs1Δ* cells (KT2919) undergoing meiotic prophase (120 min in the Supplementary Fig. S1B, right panel) were high-pressure frozen and processed for immuno EM analysis with Hrs1 antisera (4030 final bleed). Two serial sections of one SPB are presented. The arrow indicates the SPB. MT : microtubules, NE : nuclear membrane. Scale bar, 500 nm.

A

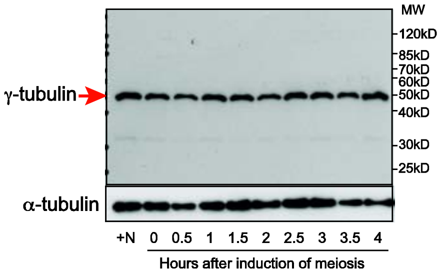

B γ-tubulin immuno labelling on serial sections of a SPB in a vegetatively growing interphase cell

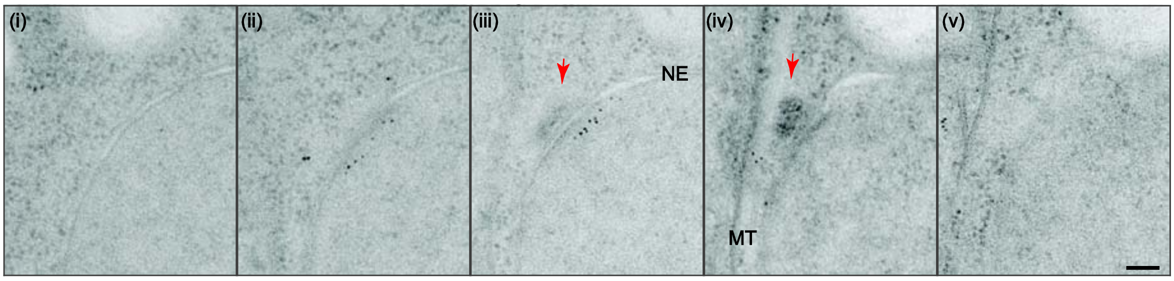

Figure S2. Validation of Anti γ-Tubulin Monoclonal Antibody, Related to Figure 3

(A) Whole cell lysates of synchronous *hrs1*$^{+}$ cells undergoing meiosis as presented in Fig. 1A were subjected to SDS-PAGE and Western blotting with the anti γ-tubulin monoclonal antibody GTU-88 (SIGMA). "+N" indicates vegetatively growing cells in YE media with rich nitrogen source. Numbers indicate hours after induction of meiosis by temperature shift. A signal at the predicted size (51 kD) is indicated by a red arrow. (B) The anti γ-tubulin monoclonal antibody GTU-88 was used for EM with immuno-gold labeling on vegetatively growing cells. They were high-pressure frozen and processed for immuno-gold labelling. Five serial sections (i)-(v) spanning a SPB are presented. The anti γ-tubulin monoclonal antibody was detected by 10 nm colloidal gold -conjugated protein A. The red arrow indicates the SPB. MT : microtubules, NE : nuclear membrane. Scale bar, 100 nm. Localisation of γ-tubulin at the nuclear plasmic side of the SPB was observed in the sections (ii) and (iii).

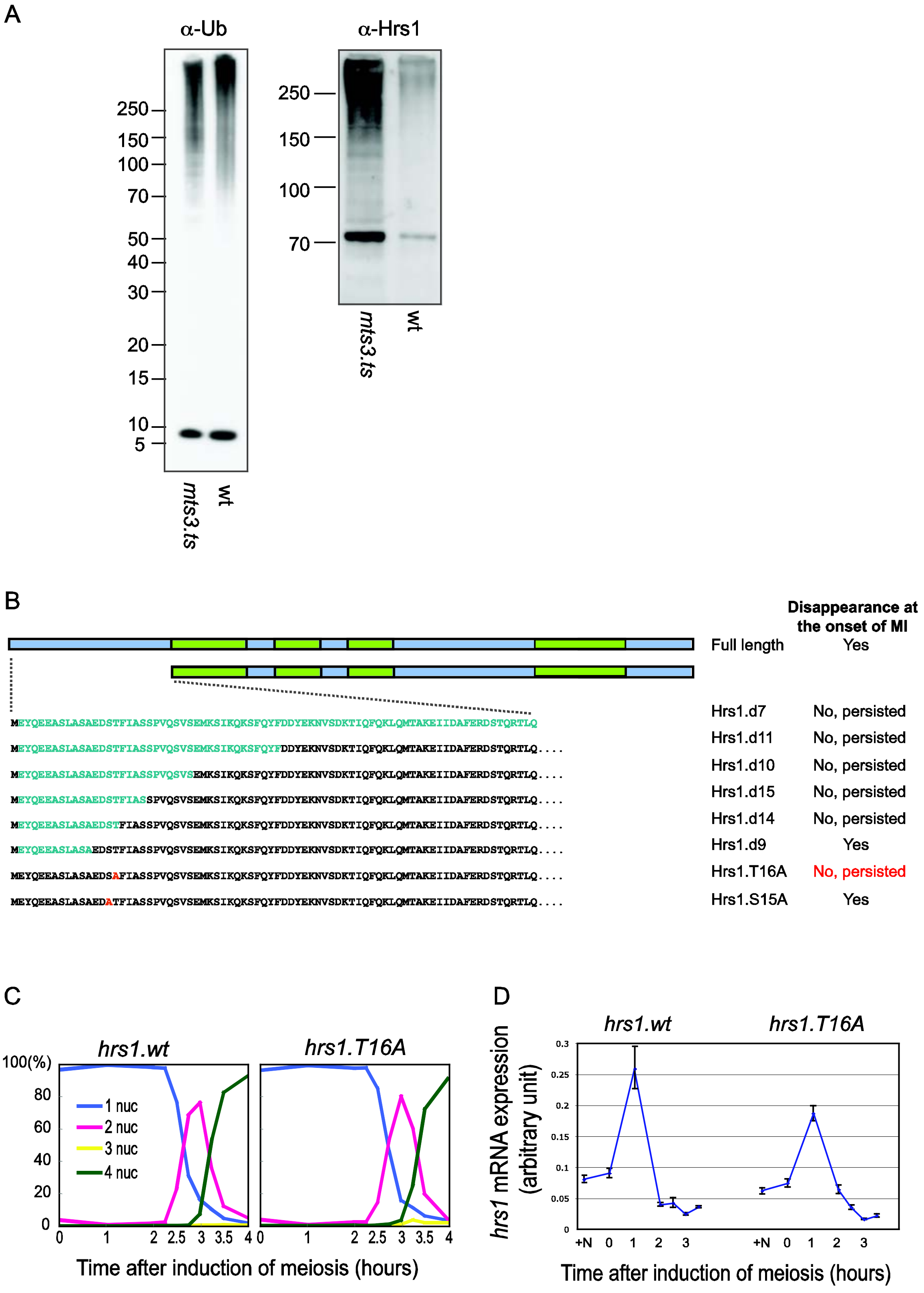
A
α-Ub
α-Hrs1
250
150
100
70
50
40
30
20
15
10
5
mts3.ts
wt
B
Disappearance at the onset of MI
Full length Yes
MEYQEEASLASAEDSTFIASSPVQSVSEMKSIKQKSFQYFDDYEKNVSDKTIQFQKLQMTAKEIIDAFERDSTQRTLQ
Hrs1.d7 No, persisted
MEYQEEASLASAEDSTFIASSPVQSVSEMKSIKQKSFQYFDDYEKNVSDKTIQFQKLQMTAKEIIDAFERDSTQRTLQ....
Hrs1.d11 No, persisted
MEYQEEASLASAEDSTFIASSPVQSVSEMKSIKQKSFQYFDDYEKNVSDKTIQFQKLQMTAKEIIDAFERDSTQRTLQ....
Hrs1.d10 No, persisted
MEYQEEASLASAEDSTFIASSPVQSVSEMKSIKQKSFQYFDDYEKNVSDKTIQFQKLQMTAKEIIDAFERDSTQRTLQ....
Hrs1.d15 No, persisted
MEYQEEASLASAEDSTFIASSPVQSVSEMKSIKQKSFQYFDDYEKNVSDKTIQFQKLQMTAKEIIDAFERDSTQRTLQ....
Hrs1.d14 No, persisted
MEYQEEASLASAEDSTFIASSPVQSVSEMKSIKQKSFQYFDDYEKNVSDKTIQFQKLQMTAKEIIDAFERDSTQRTLQ....
Hrs1.d9 Yes
MEYQEEASLASAEDSAFIASSPVQSVSEMKSIKQKSFQYFDDYEKNVSDKTIQFQKLQMTAKEIIDAFERDSTQRTLQ....
Hrs1.T16A No, persisted
MEYQEEASLASAEDATFIASSPVQSVSEMKSIKQKSFQYFDDYEKNVSDKTIQFQKLQMTAKEIIDAFERDSTQRTLQ....
Hrs1.S15A Yes
C
hrs1.wt
hrs1.T16A
100(%)
80
60
40
20
0
1 nuc
2 nuc
3 nuc
4 nuc
0 1 2 2.5 3 3.5 4
Time after induction of meiosis (hours)
D
hrs1.wt
hrs1.T16A
hrs1 mRNA expression (arbitrary unit)
0.3
0.25
0.2
0.15
0.1
0.05
0
+N 0 1 2 3
Time after induction of meiosis (hours)

Figure S3. Related to Figure 4

(A) Hrs1 is subjected to Ubiquitilation. 6His-ubiquitin (pREP1-6His-Ub) was expressed in wild type or *mts3.ts* cells harboring *hrs1-GFP-2xFLAG*. Cells were induced for meiosis at a semi-permissive temperature (30°C) and ubiquitylated proteins were purified with Ni-NTA beads. Samples containing comparable amount of mono-ubiquitin were loaded (left panel). Immunoblotting was performed with anti-Hrs1 antibodies to examine ubiquitilation of Hrs1 (right panel).

(B) A diagram to depict *hrs1* N-terminal truncation alleles. Amino acid residues are presented in one letter abbreviation. Residues presented in blue are missing in the indicated *hrs1* mutant alleles. Green boxes represent predicted coiled-coil motifs [15]. Each allele was tagged with 2xFLAG-GFP, integrated at the *hrs1* gene locus and the GFP signal observed at the transition from meiotic prophase to MI. Disappearance of the GFP signal occurred in the strain harboring the *hrs1.d9* allele but not in the *hrs1.d14* strain indicating that E(13)-D(14)-S(15)-T(16) contains the crucial residue(s) for Hrs1 destruction. An alanine substitution of T16 (T16A mutation) caused Hrs1 to be stabilized.

(C) and (D) : The *hrs1.T16A* transcript level is comparable to the one of *hrs1.wt*. Cells of h^{-}/h^{-} *pat1.114/pat1.114 mat-Pc hrs1.wt-GFP-2xFLAG/hrs1.wt-GFP-2xFLAG* and h^{-}/h^{-} *pat1.114/pat1.114 mat-Pc hrs1.T16A-GFP-2xFLAG/hrs1.T16A-GFP-2xFLAG* were synchronised at G1 by nitrogen starvation and were induced for meiotic differentiation by temperature shift. Synchronicity was monitored by counting the number of nuclei (C). Total RNAs were isolated at the time indicated and cDNAs were prepared for quantitative PCR (qPCR) reactions to estimate the *hrs1.wt-GFP* and *hrs1.T16A-GFP* transcripts levels (D). “+N” indicates vegetatively growing cells. The *act1* gene expression level was used as an internal control to normalize the *hrs1* transcript expression level. Quadplex samples were used to deduce the average expression level at each time point.

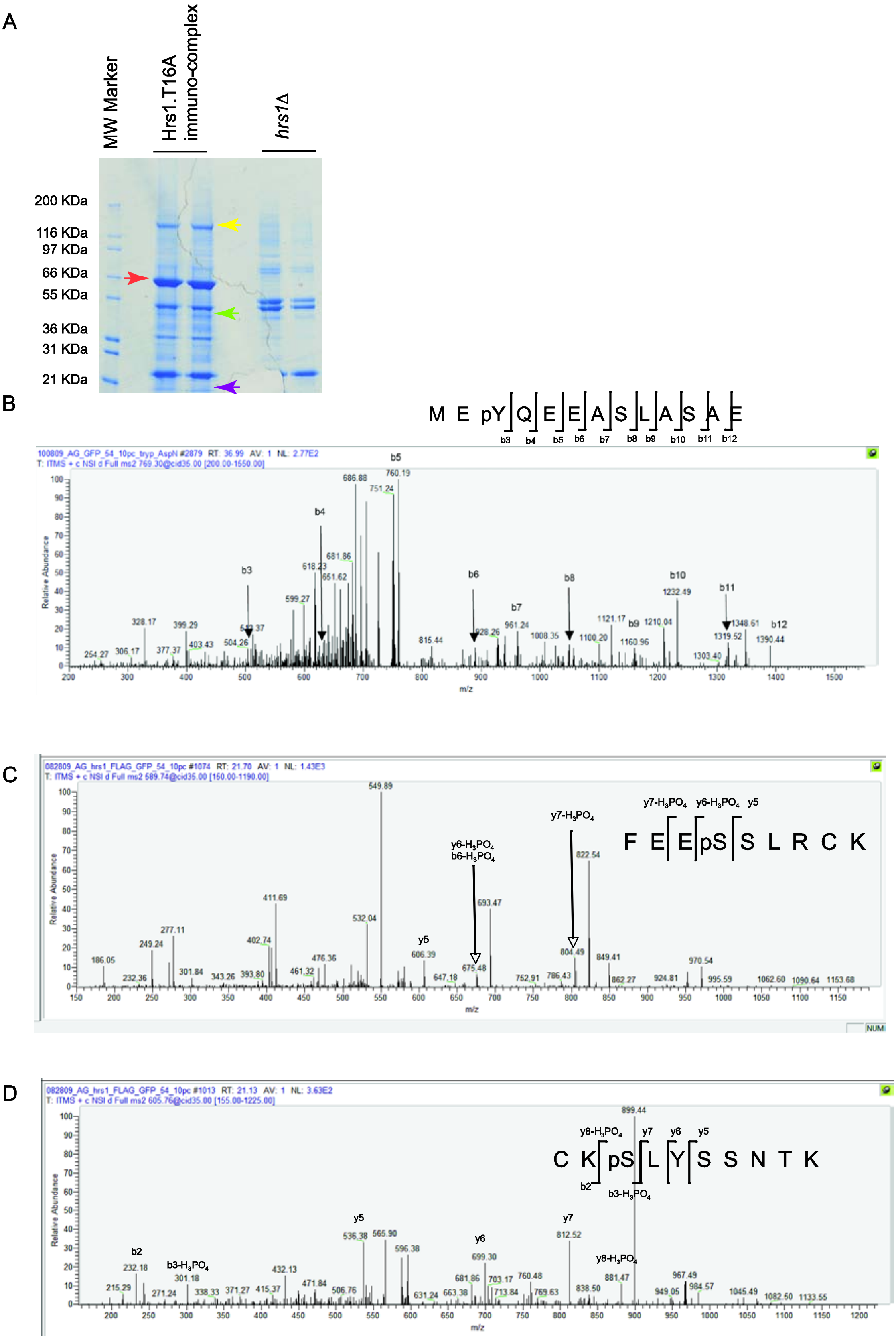
A
MW Marker
Hrs1.T16A immuno-complex
hrs1Δ
200 KDa
116 KDa
97 KDa
66 KDa
55 KDa
36 KDa
31 KDa
21 KDa
B
M E pY Q E E A S L A S A E
b3 b4 b5 b6 b7 b8 b9 b10 b11 b12
Relative Abundance
m/z
C
F E E pS S L R C K
y7-H3PO4 y6-H3PO4 y5
y6-H3PO4
b6-H3PO4
Relative Abundance
m/z
D
C K pS L Y S S N T K
y8-H3PO4 y7 y6 y5
b2 b3-H3PO4
Relative Abundance
m/z

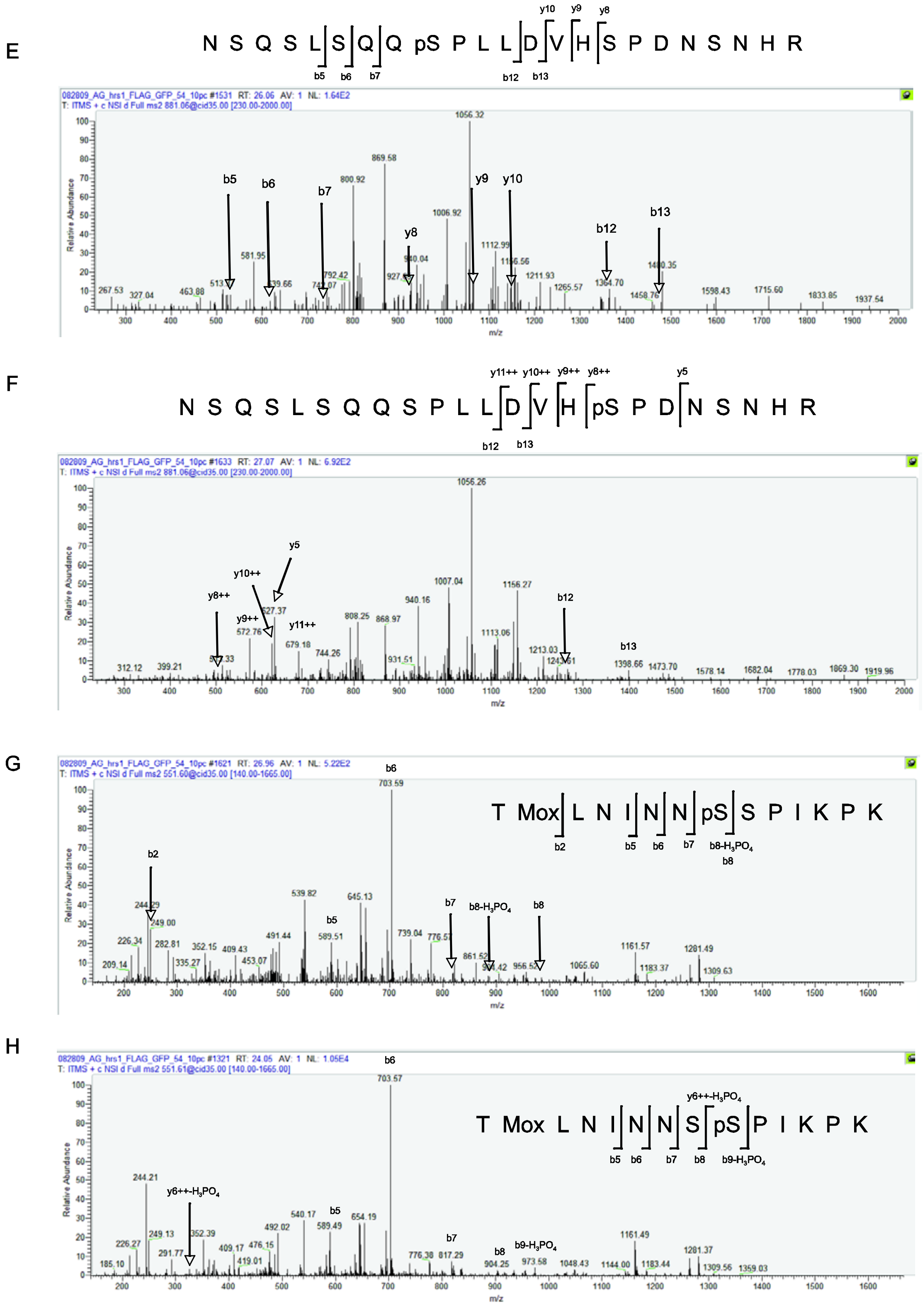
E
N S Q S L S Q Q pS P L L D V H S P D N S N H R
y10 y9 y8
b5 b6 b7 b12 b13
082809_AG_hrs1_FLAG_GFP_54_10pc #1531 RT: 26.06 AV: 1 NL: 1.64E2
T: ITMS + c NSI d Full ms2 881.06@cid35.00 [230.00-2000.00]
Relative Abundance
m/z
F
N S Q S L S Q Q S P L L D V H pS P D N S N H R
y11++ y10++ y9++ y8++ y5
b12 b13
082809_AG_hrs1_FLAG_GFP_54_10pc #1633 RT: 27.07 AV: 1 NL: 6.92E2
T: ITMS + c NSI d Full ms2 881.06@cid35.00 [230.00-2000.00]
Relative Abundance
m/z
G
082809_AG_hrs1_FLAG_GFP_54_10pc #1621 RT: 26.96 AV: 1 NL: 5.22E2
T: ITMS + c NSI d Full ms2 551.60@cid35.00 [140.00-1665.00]
T Mox L N I N N pS S P I K P K
b2 b5 b6 b7 b8-H3PO4 b8
Relative Abundance
m/z
H
082809_AG_hrs1_FLAG_GFP_54_10pc #1321 RT: 24.05 AV: 1 NL: 1.05E4
T: ITMS + c NSI d Full ms2 551.61@cid35.00 [140.00-1665.00]
y6++-H3PO4
T Mox L N I N N S pS P I K P K
b5 b6 b7 b8 b9-H3PO4
Relative Abundance
m/z

Figure S4-1 and S4-2. Identification of Hrs1 Phosphorylation Sites, Related to Figures 4 and 5

(A) A Coomassie stained SDS-PAGE gel image of the Hrs1.T16A immunocomplex prepared from diploid *pat1.114 mat-Pc hrs1.wt-2xFLAG-GFP* cells undergoing transition from meiotic prophase to MI (135 min after the induction of meiosis). The Hrs1.T16A-2xFLAG-GFP, indicated by a red arrow, was missing in the sample prepared in the exact same manner from a strain (KT2919) harboring *hrs1* deletion gene allele (right two lanes). Identified associated proteins that interact with Hrs1 are indicated by arrows; yellow arrow: Mto1, green arrow: Kms2, purple arrow: Cdc31. The Hrs1.T16A-2xFLAG-GFP (red arrow) was excised from the gel and subjected to LC MS/MS analyses. Results are presented in Supplementary Fig. S4(B)-(H).

(B)-(H) Product ion spectrum from Hrs1.T16A-2xFLAG-GFP with annotated peptide sequence. Pertinent diagnostic ions labelled. (B) Site of phosphorylation localised to Y3 by continual b ion series from b3 through to b12. (C) Site of phosphorylation localised to S152 by y5, y6-H_3PO_4/b6-H_3PO_4 and y7-H_3PO_4.

(D) Site of phosphorylation localised to S158 by both y8-H_3PO_4 and b3-H_3PO_4.

(E) Site of phosphorylation localised to S209 by b5, b6,b7 b12, b13 and y8-y10.

(F) Site of phosphorylation localised to S216 by y8++,y9++,y10++,y11++,b13 and y5.

(G) Site of phosphorylation localised to S231 by both b8-H_3PO_4 and b8. (H) Site of phosphorylation localised to S232 by both y6++-H_3PO_4 and b9-H_3PO_4.

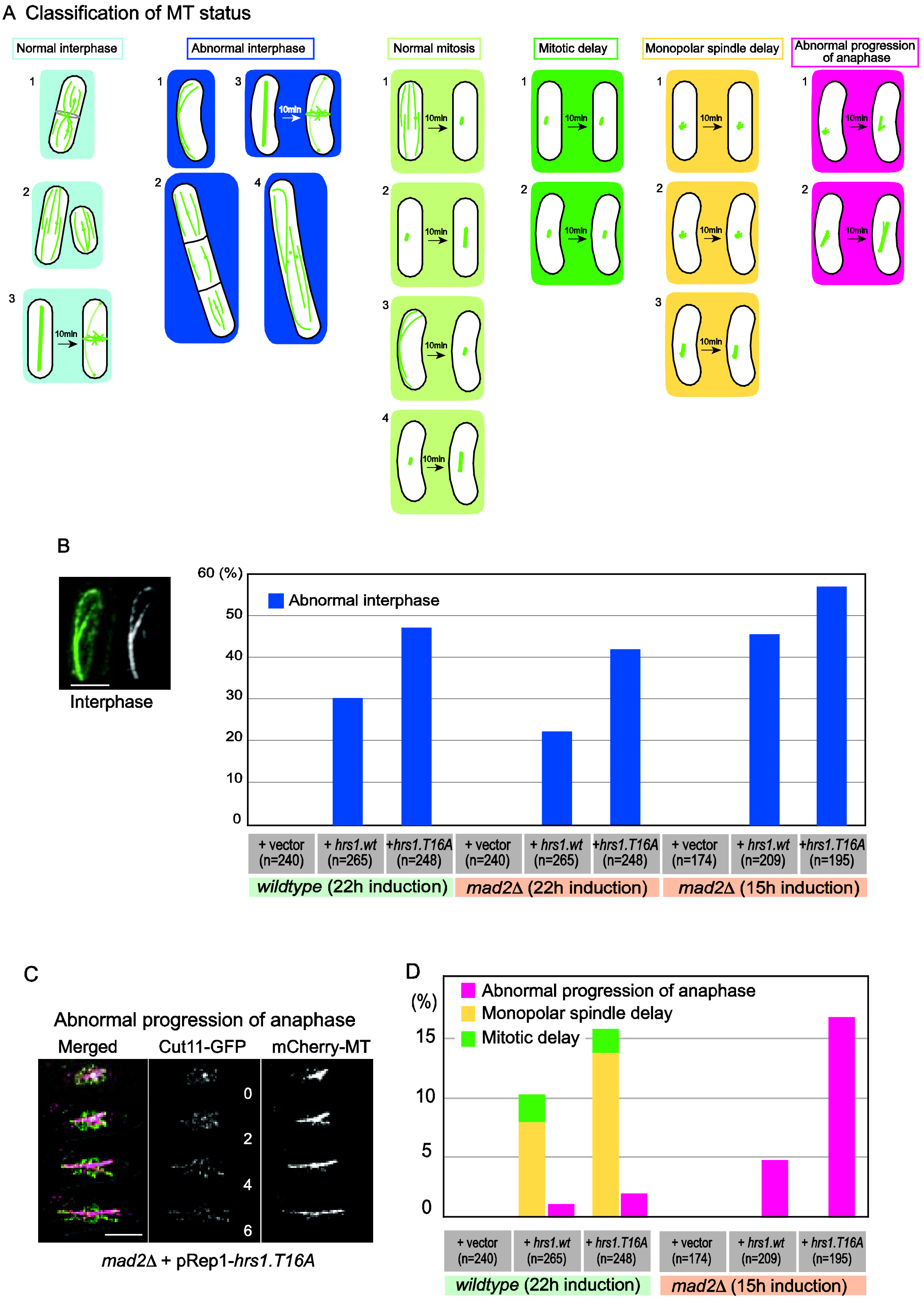
A Classification of MT status
Normal interphase
Abnormal interphase
Normal mitosis
Mitotic delay
Monopolar spindle delay
Abnormal progression of anaphase
10min
B
Interphase
60 (%)
Abnormal interphase
50
40
30
20
10
0
+ vector (n=240)
+ hrs1.wt (n=265)
+hrs1.T16A (n=248)
+ vector (n=240)
+ hrs1.wt (n=265)
+hrs1.T16A (n=248)
+ vector (n=174)
+ hrs1.wt (n=209)
+hrs1.T16A (n=195)
wildtype (22h induction)
mad2Δ (22h induction)
mad2Δ (15h induction)
C
Abnormal progression of anaphase
Merged
Cut11-GFP
mCherry-MT
0
2
4
6
mad2Δ + pRep1-hrs1.T16A
D
(%)
Abnormal progression of anaphase
Monopolar spindle delay
Mitotic delay
15
10
5
0
+ vector (n=240)
+ hrs1.wt (n=265)
+ hrs1.T16A (n=248)
+ vector (n=174)
+ hrs1.wt (n=209)
+ hrs1.T16A (n=195)
wildtype (22h induction)
mad2Δ (15h induction)

Figure S5. Related to Figure 6

(A) Classification of the MT phenotypes used in the Supplementary Fig. S5B and D. MT arrangements were classified as follows : “Normal interphase”: 1. interphase cells with septa, 2. interphase cells without cepta, 3. cells travel through telophase to G1 phase, “Abnormal interphase”: 1. interphase cells with rMT arrangement. Such cells are often banana-shaped, 2. interphase cells with multiple septa, 3. banana-shaped cells travelling through telophase to G1 phase, 4. very long interphase cells with aberrant MT structure, “Normal mitosis”: 1. cells at the transition of G2 to M, 2. cells at the transition of anaphase A to B, 3. banana-shaped cells at the transition of G2 to M, 4. banana-shaped cells at the transition of anaphse A to B, “Mitotic delay”: 1 and 2. regardless of the cell shape, cells with constant spindle length throughout the 10 min window of the filming, “Monopolar spindle delay”: regardless of the cell shape, cells with monopolar spindles of constant spindle length throughout the 10 min window of the filming and “Abnormal progression of anaphse”: regardless of the cell shape, cells with aberrant spindles which elongate. Cells with elongating monopolar spindles are in this category. Importantly, we defined “cells delayed in mitosis” as cells with a constant spindle length throughout the 10 min of filming. This is because the duration of “anaphase A” is less than 10 min at 25-30°C [2] and we expected cells with unperturbed spindle formation to transit from anaphase A to anaphase B (when spindle elongation occurs) during this time window.

(B) (Left panel) A representative image of the rMT-like aberrant structure of interphase MT generated in a cell ectopically expressing the Hrs1.T16A mutant protein. GFP-α-tubulin signal was recorded. (Right panel) Summary of the populations of cells with abnormal interphase MT when Hrs1.wt and Hrs1.T16A were ectopically expressed in the wild type or *Δmad2* cells.

(C) The elongating monopolar spindle structure in a *mad2* deletion cell was encapsulated by the GFP signal of the Cut11-GFP, a SPB and nuclear envelope marker [16]. Hrs1.T16A expression was induced for 15 hours in a *Δmad2* cell carrying mCherry-Atb2 (α-tubulin) and Cut11-GFP. Fluorescent signals were recorded at 1minute intervals using a confocal microscope with simultaneous excitation and recording of both colors. Scale bar, 5 μm.

(D) Summary of *hrs1* overexpression mitotic phenotype. pRep1-*hrs1.wt* or pRep1-*hrs1.T16A* were introduced into wild type and *Δmad2* cells harboring GFP-Atb2. *hrs1.wt* or *hrs1.T16A* were induced for 22 hours (in wild type cells) or 15 hours (in *Δmad2* cells) as prolonged expression of Hrs1 in *Δmad2* cells resulted in an accumulation of interphase cells and inviable cell remains with few mitotic cells. The GFP-Atb2 signals were captured for 10 mins at 1minute intervals. 15-30 serial images of 0.5 μm interval along the Z axis were taken at each time point to span the full thickness of the cells. Deconvolved and Z-projected images were used for analysis. Cells were classified for their MT structure according to the diagram depicted in Supplementary Fig. S5A. Population of the cells in the each category is presented.

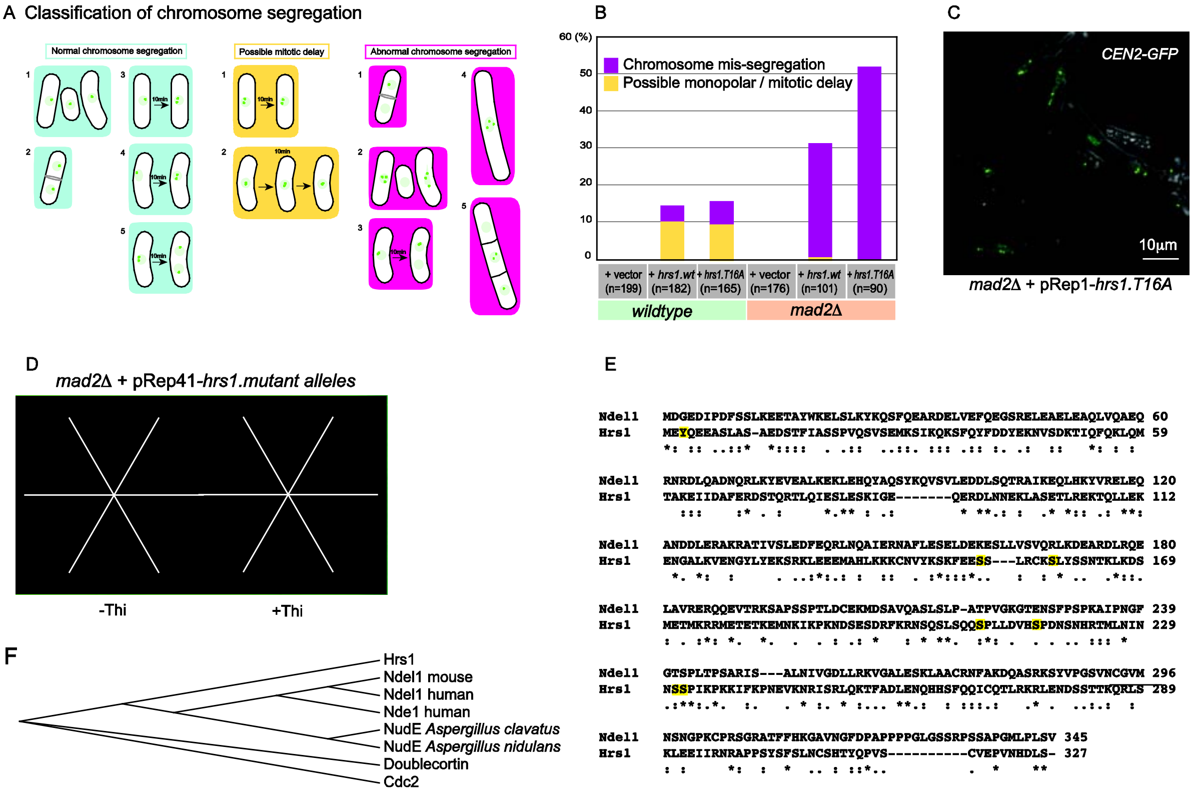
A Classification of chromosome segregation
Normal chromosome segregation
Possible mitotic delay
Abnormal chromosome segregation
10min
B
60 (%)
50
40
30
20
10
0
Chromosome mis-segregation
Possible monopolar / mitotic delay
+ vector (n=199)
+ hrs1.wt (n=182)
+ hrs1.T16A (n=165)
+ vector (n=176)
+ hrs1.wt (n=101)
+ hrs1.T16A (n=90)
wildtype
mad2Δ
C
CEN2-GFP
10μm
mad2Δ + pRep1-hrs1.T16A
D
mad2Δ + pRep41-hrs1.mutant alleles
-Thi
+Thi
E
Ndel1 MDGEDIPDFSSLKEETAYWKELSLKYKQSFQEARDELVEFQEGSRELEAELEAQLVQAEQ 60
Hrs1 MEYQEEASLAS-AEDSTFIASSPVQSVSEMKSIKQKSFQYFDDYEKNVSDKTIQFQKLQM 59
Ndel1 RNRDLQADNQRLKYEVEALKEKLEHQYAQSYKQVSVLEDDLSQTRAIKEQLHKYVRELEQ 120
Hrs1 TAKEIIDAFERDSTQRTLQIESLESKIGE--------QERDLNNEKLASETLREKTQLLEK 112
Ndel1 ANDDLERAKRATIVSLEDFEQRLNQAIERNAFLESELDEKESLLVSVQRLKDEARDLRQE 180
Hrs1 ENGALKVENGYLYEKSRKLEEEMAHLKKKCNVYKSKFEESS---LRCKSLYSSNTKLKDS 169
Ndel1 LAVRERQQEVTRKSAPSSPTLDCEKMDSAVQASLSLP-ATPVGKGTENSFPSPKAIPNGF 239
Hrs1 METMKRRMETETKEMNKIKPKNDSESDRFKRNSQSLSQQSPLLDVHSPDNSNHRTMLNIN 229
Ndel1 GTSPLTPSARIS---ALNIVGDLLRKVGALESKLAACRNFAKDQASRKSYVPGSVNCGVM 296
Hrs1 NSSPIKPKKIFKPNEVKNRISRLQKTFADLENQHHSFQQICQTLRKRLENDSSTTKQRLS 289
Ndel1 NSNGPKCPRSGRATFFHKGAVNGFDPAPPPPGLGSSRPSSAPGMLPLSV 345
Hrs1 KLEEIIRNRAPPSYSFSLNCSHTYQPVS----------CVEPVNHDLS- 327
F
Hrs1
Ndel1 mouse
Ndel1 human
Nde1 human
NudE Aspergillus clavatus
NudE Aspergillus nidulans
Doublecortin
Cdc2

Figure S6. Related to Figure 6

(A) Classification of the CEN2-GFP segregation phenotypes used in Supplementary Fig. S6B.

(B) *hrs1* overexpression induces chromosome mis-segregation. Wild type and Δ*mad2* cells, harbouring GFP-marked centromere 2 (CEN2-GFP), were introduced with either pRep1-*hrs1.wt* or pRep1-*hrs1.T16A* and their expression induced for 22 hours before the CEN2-GFP signal was recorded. Imaging was performed for 10 min at 1 minute intervals. 15-30 serial images of 0.5 μm interval along the Z axis were taken at each time point to span the full thickness of the cells. Deconvolved and Z-projected images were used for analysis.

(C) A typical image of CEN2-GFP in *Δmad2* cells overexpressing *hrs1.T16A*. Cell morphology and CEN2-GFP distribution became heavily heterogenous after induction of *hrs1.T16A* for 22 hours. 17 serial images of 0.5-0.6 μm interval along the Z axis were taken to span the full thickness of the cell. Deconvolved and Z-projected images are presented. Scale bar, 10 μm.

(D) Toxicity of the ectopic Hrs1 expression during the vegetative cell cycle in *mad2Δ* cells is dependent on the Hrs1 modification status. Various *hrs1* mutants, as well as wild type *hrs1*, were induced under the *nmt41* promoter in *mad2Δ* cells and their ability to interfere with cell growth was assessed on a plate containing Phloxin B, a dye which penetrates into inviable cells. The patch of cells expressing Hrs1.T16A (*T16A*), a stabilised mutant, showed darker pink color compared to the cells expressing wild type Hrs1 (*wt*) in agreement with the result obtained with a stronger *nmt1* promoter presented in Figs. S6B and S6C. Among all the mutant *hrs1* alleles tested, *hrs1.T16A-FA6*, a stabilised and non-phosphorylatable mutant (*T16A-FA6*), was most effective in interfering cell viability, giving the darkest pink colour. *hrs1.D7*, a phosphomimetic mutant (*D7*), hardly showed any interference, while *hrs1.FA6*, a non-phosphorylatable mutant (*FA6*), showed a severe growth interference.

(E) Hrs1 shows similarity with Ndel1. Mouse Ndel1 and Hrs1 primary sequences are compared using ClustalW [17]. Hrs1 residues which receive phosphorylation during meiotic differentiation are highlighted in yellow.

(F) Phylogenetic tree of NudE family proteins and Hrs1. Mouse Ndel1, human Ndel1, human Nde1, *Aspergillus clavatus* NudE, *Aspergillus nidulans* NudE and Hrs1 were subject to ClustalW along with negative controls (Cdc2 (mitotic kinase of similar molecular weight) and Doublecortin (another causal protein for lissencephaly and involved in MT regulation). Phylogenetic tree was drawn by Treeview software based on ClustalW analysis [17].

Table S1 . Strains Used in This Study

Figure 1

KT2486 h^{-}/h^{-} pat1.114/pat1.114 ade6.M216/ade6.M210 leu1.32/leu1.32 lys1^{+}/lys1::mat-Pc-lys1^{+}

KT2919 h^{-}/h^{-} pat1.114/pat1.114 ade6.M216/ade6.M210 leu1.32/leu1.32 lys1^{+}/lys1::mat-Pc-lys1^{+} Δhrs1::Hyg^{r}/Δhrs1::Hyg^{r}

Figure 2

KT2486 h^{-}/h^{-} pat1.114/pat1.114 ade6.M216/ade6.M210 leu1.32/leu1.32 lys1^{+}/lys1::mat-Pc-lys1^{+}

KT2919 h^{-}/h^{-} pat1.114/pat1.114 ade6.M216/ade6.M210 leu1.32/leu1.32 lys1^{+}/lys1::mat-Pc-lys1^{+} Δhrs1::Hyg^{r}/Δhrs1::Hyg^{r}

Figure 3

(A)

KT2486 h^{-}/h^{-} pat1.114/pat1.114 ade6.M216/ade6.M210 leu1.32/leu1.32 lys1^{+}/lys1::mat-Pc-lys1^{+}

(D)

KT2919 h^{-}/h^{-} pat1.114/pat1.114 ade6.M216/ade6.M210 leu1.32/leu1.32 lys1^{+}/lys1::mat-Pc-lys1^{+} Δhrs1::Hyg^{r}/Δhrs1::Hyg^{r}

Figure 4

(A)

KT2901 h^{90} hrs1-GFP-2xFLAG::Kan^{r} Pnda3-mCherry-atb2<<aur1^{r} ade6.M216 leu1.32

(B)

KT3325 h^{90} mts3.1^{ts} hrs1-GFP-2xFLAG<<Kan^{r} Pnda3-mCherry-atb2<<aur1^{r} ade6.M216 leu1.32

(C)

KT2902 h^{90} hrs1.T16A-GFP-2xFLAG<<Kanr Pnda3-mCherry-atb2<<aur1^{r} ade6.M216 leu1.32

(D)

KT2469 h^{-}/h^{-} pat1.114/pat1.114 ade6.M216/ade6.M210 leu1.32/leu1.32 lys1^{+}/lys1::mat-Pc-lys1^{+} hrs1-GFP-2xFLAG<<Kan^{r}/hrs1-GFP-2xFLAG<<Kan^{r}

KT2474 h^{-}/h^{-} pat1.114/pat1.114 ade6.M216/ade6.M210 leu1.32/leu1.32 lys1^{+}/lys1::mat-Pc-lys1^{+} hrs1.T16A-GFP-2xFLAG<<Kan^{r}/hrs1.T16A-GFP-2xFLAG<<Kan^{r}

(E)

KT2474 h^{-}/h^{-} pat1.114/pat1.114 ade6.M216/ade6.M210 leu1.32/leu1.32 lys1^{+}/lys1::mat-Pc-lys1^{+} hrs1.T16A-GFP-2xFLAG<<Kan^{r}/hrs1.T16A-GFP-2xFLAG<<Kan^{r}

(G)

KT3302 h^{-}/h^{-} pat1.114/pat1.114 ade6.M216/ade6.M210 leu1.32/leu1.32 lys1^{+}/lys1::mat-Pc-lys1^{+} hrs1.T16A-FA6-GFP-2xFLAG<<Kan^{r}/hrs1.T16A-FA6-GFP-2xFLAG<<Kan^{r}

KT2474 h^{-}/h^{-} pat1.114/pat1.114 ade6.M216/ade6.M210 leu1.32/leu1.32 lys1^{+}/lys1::mat-Pc-lys1^{+} hrs1.T16A-GFP-2xFLAG<<Kan^{r}/hrs1.T16A-GFP-2xFLAG<<Kan^{r}

Figure 5

(A)

KT3156 h^{90} *ade6.M210 leu1.32 Pnda3-mCherry-atb2<<aur1*^{r} *hrs1.T.16A-FA6-GFP-2xFLAG<<Kan*^{r}

(B)

KT3553 h^{90} *ade6.M210 leu1.32 Pnda3-mCherry-atb2<<aur1*^{r} *hrs1.D7-GFP-2xFLAG<<Kan*^{r}

(D)

KT3156 h^{90} *ade6.M210 leu1.32 Pnda3-mCherry-atb2<<aur1*^{r} *hrs1.T16A-FA6-GFP-2xFLAG<<Kan*^{r}

(E)

KT3156 h^{90} *ade6.M210 leu1.32 Pnda3-mCherry-atb2<<aur1*^{r} *hrs1.T16A-FA6-GFP-2xFLAG<<Kan*^{r}

KT3182 h^{90} *ade6.M210 leu1.32 Pnda3-mCherry-atb2<<aur1*^{r} *hrs1.T16A-FA6-GFP-2xFLAG<<Kan*^{r} Δ*mad2::ClonNAT*

KT2901 h^{90} *ade6.M216 leu1.32 Pnda3-mCherry-atb2<<aur1*^{r} *hrs1-GFP-2xFLAG<<Kanr*

(F)

KT3159 h^{90} *ade6.M210 leu1.32 ura4.d18 hrs1.T16A-FA6-GFP-2xFLAG<<Kan*^{r} Δ*mad2::ura4*$^{+}$ *Pnda3-mCherry-atb2<<aur1*^{r}

(G)

KT3327 h^{90} *ade6.M216 leu1.32 hrs1.wt*$^{+}$*<<nat*^{r}

KT3328 h^{90} *ade6.M216 leu1.32 hrs1.wt*$^{+}$*<<nat*^{r}

KT3266 h^{90} *ade6.M216 leu1.32* Δ*hrs1::Hyg*^{r}

KT3267 h^{90} *ade6.M216 leu1.32* Δ*hrs1::Hyg*^{r}

KT3330 h^{90} *ade6.M216 leu1.32 hrs1.T16A<<nat*^{r}

KT3331 h^{90} *ade6.M216 leu1.32 hrs1.T16A<<nat*^{r}

KT3332 h^{90} *ade6.M216 leu1.32 hrs1.T16A-FA6<<nat*^{r}

KT3333 h^{90} *ade6.M216 leu1.32 hrs1.T16A-FA6<<nat*^{r}

KT3335 h^{90} *ade6.M216 leu1.32 hrs1.FA6<<nat*^{r}

KT3336 h^{90} *ade6.M216 leu1.32 hrs1.FA6<<nat*^{r}

KT3274 h^{90} *ade6.M210 leu1.32 ura4.d18 hrs1.wt*$^{+}$*<<nat*^{r} Δ*mad2::ura4*$^{+}$

KT3276 h^{90} *ade6.M210 leu1.32 ura4.d18 hrs1.wt*$^{+}$*<<nat*^{r} Δ*mad2::ura4*$^{+}$

KT1841 h^{90} *ade6.M210 leu1.32 ura4.d18* Δ*hrs1::ura4*$^{+}$ Δ*mad2::ura4*$^{+}$

KT1842 h^{90} *ade6.M210 leu1.32 ura4.d18* Δ*hrs1::ura4*$^{+}$ Δ*mad2::ura4*$^{+}$

KT3278 h^{90} *ade6.M210 leu1.32 ura4.d18 hrs1.T16A<<nat*^{r} Δ*mad2::ura4*$^{+}$

KT3279 h^{90} *ade6.M210 leu1.32 ura4.d18 hrs1.T16A<<nat*^{r} Δ*mad2::ura4*$^{+}$

KT3283 h^{90} *ade6.M210 leu1.32 ura4.d18 hrs1.T16A-FA6<<nat*^{r} Δ*mad2::ura4*$^{+}$

KT3284 h^{90} *ade6.M210 leu1.32 ura4.d18 hrs1.T16A-FA6<<nat*^{r} Δ*mad2::ura4*$^{+}$

KT3287 h^{90} *ade6.M210 leu1.32 ura4.d18 hrs1.FA6<<nat*^{r} Δ*mad2::ura4*$^{+}$

KT3288 h^{90} *ade6.M210 leu1.32 ura4.d18 hrs1.FA6<<nat*^{r} Δ*mad2::ura4*$^{+}$

(H)

KT3182 *h*^{90} *ade6.M210 leu1.32 Pnda3-mCherry-atb2<<aur1*^{r} *hrs1.T16A-FA6-GFP-2xFLAG<<Kan*^{r} *Δmad2::ClonNAT*

Figure 6

(A)

KT2486 *h*$^{-}$*/h*$^{-}$ *pat1.114/pat1.114 ade6.M216/ade6.M210 leu1.32/leu1.32 lys1*$^{+}$*/lys1::mat-Pc-lys1*$^{+}$

(B)

KT2510 *h*^{90} *ade6.M216 leu1.32 Pnda3-GFP-atb2<<aur1*^{r} + *pRep1-hrs1.T16A-HA*

(C)

KT2982 *h*^{90} *ade6.M210 leu1.32 Pnda3-mCherry-atb2<<aur1*^{r} *sid4-GFP<<Hyg*^{r} + *pRep1-hrs1.T16A-HA*

(D)

KT3860 *h*^{90} *ade6.M210 leu1.32 Pnda3-mCherry-atb2<<aur1*^{r} +pRep1-T16A-FA6-HA

Figure S1

(B)

KT2486 *h*$^{-}$*/h*$^{-}$ *pat1.114/pat1.114 ade6.M216/ade6.M210 leu1.32/leu1.32 lys1*$^{+}$*/lys1::mat-Pc-lys1*$^{+}$

KT2919 *h*$^{-}$*/h*$^{-}$ *pat1.114/pat1.114 ade6.M216/ade6.M210 leu1.32/leu1.32 lys1*$^{+}$*/lys1::mat-Pc-lys1*$^{+}$ *Δhrs1::Hyg*^{r}*/Δhrs1::Hyg*^{r}

(C)

KT2919 *h*$^{-}$*/h*$^{-}$ *pat1.114/pat1.114 ade6.M216/ade6.M210 leu1.32/leu1.32 lys1*$^{+}$*/lys1::mat-Pc-lys1*$^{+}$ *Δhrs1::Hyg*^{r}*/Δhrs1::Hyg*^{r}

Figure S2

(A and B)

KT2486 *h*$^{-}$*/h*$^{-}$ *pat1.114/pat1.114 ade6.M216/ade6.M210 leu1.32/leu1.32 lys1*$^{+}$*/lys1::mat-Pc-lys1*$^{+}$

Figure S3

(A)

KT3914 *h*^{90} *hrs1-GFP-2xFLAG<<Kanr ade6.M216 leu1.32* + pRep1-His6-Ub

KT3917 *h*^{90} *mts3.1 hrs1-GFP-2xFLAG<<Kanr ade6.M216 leu1.32* + pRep1-His6-Ub

(C and D)

KT2469 *h*$^{-}$*/h*$^{-}$ *pat1.114/pat1.114 ade6.M216/ade6.M210 leu1.32/leu1.32 lys1*$^{+}$*/lys1::mat-Pc-lys1*$^{+}$ *hrs1-GFP-2xFLAG<<Kan*^{r}*/hrs1-GFP-2xFLAG<<Kan*^{r}

KT2474 *h*$^{-}$*/h*$^{-}$ *pat1.114/pat1.114 ade6.M216/ade6.M210 leu1.32/leu1.32 lys1*$^{+}$*/lys1::mat-Pc-lys1*$^{+}$ *hrs1.T16A-GFP-2xFLAG<<Kan*^{r}*/hrs1.T16A-GFP-2xFLAG<<Kan*^{r}

Figure S4-1 and S4-2

(A)

KT2474 h$^-$/h$^-$ pat1.114/pat1.114 ade6.M216/ade6.M210 leu1.32/leu1.32 lys1$^+$/lys1::mat-Pc-lys1$^+$ hrs1.T16A-GFP-2xFLAG<<Kan^r/hrs1.T16A-GFP-2xFLAG<<Kan^r

KT2919 h$^-$/h$^-$ pat1.114/pat1.114 ade6.M216/ade6.M210 leu1.32/leu1.32 lys1$^+$/lys1::mat-Pc-lys1$^+$ Δhrs1::Hyg^r/Δhrs1::Hyg^r

(B–H)

KT2474 h$^-$/h$^-$ pat1.114/pat1.114 ade6.M216/ade6.M210 leu1.32/leu1.32 lys1$^+$/lys1::mat-Pc-lys1$^+$ hrs1.T16A-GFP-2xFLAG<<Kan^r/hrs1.T16A-GFP-2xFLAG<<Kan^r

Figure S5

(B)

KT2504 h^{90} ade6.M216 leu1.32 Pnda3-GFP-atb2<<aur1^r + pRep1-empty

KT2507 h^{90} ade6.M216 leu1.32 Pnda3-GFP-atb2<<aur1^r + pRep1-hrs1.wt-HA

KT2510 h^{90} ade6.M216 leu1.32 Pnda3-GFP-atb2<<aur1^r + pRep1-hrs1.T16A-HA

KT2531 h^{90} ade6.M216 leu1.32 Pnda3-GFP-atb2<<aur1^r Δmad2::nat^r + pRep1-empty

KT2534 h^{90} ade6.M216 leu1.32 Pnda3-GFP-atb2<<aur1^r Δmad2::nat^r + pRep1-hrs1.wt-HA

KT2537 h^{90} ade6.M216 leu1.32 Pnda3-GFP-atb2<<aur1^r Δmad2::nat^r + pRep1-hrs1.T16A-HA

(C)

KT3580 h^{90} hrs1-GFP-2xFLAG<<Kan^r Pnda3-mCherry-atb2<<aur1^r ade6.M216 leu1.32 Δmad2::nat^r cut11-GFP<<Hyg^r + pRep1-hrs1.TA-HA

(D)

KT2504 h^{90} ade6.M216 leu1.32 Pnda3-GFP-atb2<<aur1^r + pRep1-empty

KT2507 h^{90} ade6.M216 leu1.32 Pnda3-GFP-atb2<<aur1^r + pRep1-hrs1.wt-HA

KT2510 h^{90} ade6.M216 leu1.32 Pnda3-GFP-atb2<<aur1^r + pRep1-hrs1.T16A-HA

KT2531 h^{90} ade6.M216 leu1.32 Pnda3-GFP-atb2<<aur1^r Δmad2::nat^r + pRep1-empty

KT2534 h^{90} ade6.M216 leu1.32 Pnda3-GFP-atb2<<aur1^r Δmad2::nat^r + pRep1-hrs1.wt-HA

KT2537 h^{90} ade6.M216 leu1.32 Pnda3-GFP-atb2<<aur1^r Δmad2::nat^r + pRep1-hrs1.T16A-HA

Figure S6

(B)

KT2513 h$^-$ ade6.M216 leu1.32 CEN2$^+$<<lacO-Kan^r-ura4$^+$ his7$^+$<< Pdis1-GFP-lacI-NLS ura4.d18 + pRep1-empty

KT2516 h$^-$ ade6.M216 leu1.32 CEN2$^+$<<lacO-Kan^r-ura4$^+$ his7$^+$<< Pdis1-GFP-lacI-NLS ura4.d18 + pRep1-hrs1.wt-HA

KT2519 h$^-$ ade6.M216 leu1.32 CEN2$^+$<<lacO-Kan^r-ura4$^+$ his7$^+$<< Pdis1-GFP-lacI-NLS ura4.d18 + pRep1-hrs1.T16A-HA

KT2522 h$^+$ leu1.32 Δmad2::ura4 CEN2$^+$<<lacO<<Kan^r<<ura4$^+$ his7$^+$<< Pdis1-GFP-lacI-NLS + pRep1-empty

KT2525 *h$^+$ leu1.32 Δmad2::ura4 CEN2$^+$<<lacO<<Kan^r<<ura4$^+$ his7$^+$<< Pdis1-GFP-lacI-NLS + pRep1-hrs1.wt-HA*

KT2528 *h$^+$ leu1.32 Δmad2::ura4 CEN2$^+$<<lacO<<Kan^r<<ura4$^+$ his7$^+$<< Pdis1-GFP-lacI-NLS + pRep1-hrs1.T16A-HA*

(C)

KT2528 *h$^+$ leu1.32 Δmad2::ura4 CEN2$^+$<<lacO<<Kan^r<<ura4$^+$ his7$^+$<<Pdis1-GFP-lacI-NLS + pRep1-hrs1.T16A-HA*

(D)

KT3368 *h$^-$ ade6.M216 leu1 Δmad2::Kanr + pRep41-empty*

KT3369 *h$^-$ ade6.M216 leu1 Δmad2::Kanr + pRep41-empty*

KT3372 *h$^-$ ade6.M216 leu1 Δmad2::Kanr + pRep41-hrs1.wt-GFP*

KT3373 *h$^-$ ade6.M216 leu1 Δmad2::Kanr + pRep41-hrs1.wt-GFP*

KT3644 *h$^-$ ade6.M216 leu1 Δmad2::Kanr + pRep41-hrs1.T16A-GFP*

KT3645 *h$^-$ ade6.M216 leu1 Δmad2::Kanr + pRep41-hrs1.T16A-GFP*

KT3647 *h$^-$ ade6.M216 leu1 Δmad2::Kanr + pRep41-hrs1.T16A-FA6-GFP*

KT3648 *h$^-$ ade6.M216 leu1 Δmad2::Kanr + pRep41-hrs1. T16A-FA6-GFP*

KT3651 *h$^-$ ade6.M216 leu1 Δmad2::Kanr + pRep41-hrs1. FA6-GFP*

KT3653 *h$^-$ ade6.M216 leu1 Δmad2::Kanr + pRep41-hrs1. FA6-GFP*

KT3655 *h$^-$ ade6.M216 leu1 Δmad2::Kanr + pRep41-hrs1.D7-GFP*

KT3659 *h$^-$ ade6.M216 leu1 Δmad2::Kanr + pRep41-hrs1.D7-GFP*

Movies S1 and S3

KT2486 *h$^-$/h$^-$ pat1.114/pat1.114 ade6.M216/ade6.M210 leu1.32/leu1.32 lys1$^+$/lys1::mat-Pc-lys1$^+$*

Movies S2 and S4

h$^-$/h$^-$ pat1.114/pat1.114 ade6.M216/ade6.M210 leu1.32/leu1.32 lys1$^+$/lys1::mat-Pc-lys1$^+$ Δhrs1::Hyg^r/Δhrs1::Hyg^r

Movie S5

KT2901 *h^{90} hrs1-GFP-2xFLAG::Kan^r Pnda3-mCherry-atb2<<aur1^r ade6.M216 leu1.32*

Supplemental Experimental Procedures

Yeast Strains and Media

General genetic methods and media for *S.pombe* were described previously [1]. The *mts3.1* mutant is a generous gift from Colin Gordon. A chromosome-integration vector to generate the *Pnda3-GFP-atb2<<aur1*^{r} and *Pnda3-mCherry-atb2<<aur1*^{r} alleles were generous gifts from Hisahiro Masuda and Takashi Toda. To monitor chromosome segregation, the GFP-marked centromere cen2-GFP system [2, 3], generously provided by Ayumu Yamamoto and Takeshi Sakuno, was employed.

To prepare highly synchronous meiotic culture, a temperature sensitive h$^-$/h$^-$ *pat1-114/pat1-114 mat-Pc* diploid strain [3] was employed. A temperature sensitive h$^-$/h$^-$ *pat1-114/pat1-114 mat-Pc* diploid strain was cultured at 25°C in YE (without adenine) until it reached 0.8-1 x 10^{7}cells/ml. Cells were then washed by excess amount of MM-N (1% glucose, 50 mg/ml leucine) by filtration. Washed cells were resuspended in MM-N (1% glucose, 50 mg/ml leucine) at a cell density of 4 x 10^{6} cells/ml and cultured at 25°C for 6.5 hours to induce G1 arrest. The culture was then diluted with an equal volume of fresh MM-N (1% glucose, 50 mg/ml leucine), prewarmed to 34°C, and synchronous meiosis was induced by further incubation at 34°C. A small portion of the culture was saved and fixed with 70% Ethanol to monitor the synchronicity of the culture by FCM analysis.

Gene disruption, 2xFLAG-GFP and mCherry-tagging of genes was performed using the direct chromosomal integration method described previously [4, 5]. For gene disruption with hygromycin B (SIGMA) or nourseothiricin (clonNAT, Werner BioAgents), the pCR2.1-hph and pCR2.1-nat plasmids[6], generously provided by Takashi Toda and Masamitsu Sato, were used. For 2xFLAG-GFP(S65T)-tagging, the pFA6a-2xFLAG-GFP(S65T)-kanMX6 plasmid was generated by modifying the pFA6a-GFP(S65T)-kanMX6 plasmid[4] with oligonucleotides encoding 2xFLAG sequence[7]. For mCherry-tagging, the pFA6a-mCherry-kanMX6 and pFA6a-mCherry-hphMX6 were generated by replacing GFP(S65T) of pFA6a-GFP(S65T)-kanMX6 plasmid[4] or pFA6a-GFP(S65T)-hphMX6 plasmid[6] with a mCherry cassette[8], which is a generous gift from Roger Tsien.

To generate various point mutations and deletion mutations, PCR based mutagenesis was employed [9]. The *hrs1.D7* allele was generated by GENEART®. As some of the *hrs1* mutated alleles gave a sporulation defect phenotype, *hrs1* mutant strains were generated by introducing the mutated *hrs1* gene fragments by transformation, rather than crossing of the strains. Introduced mutations or deletions were confirmed by DNA sequencing (PNACL, University of Leicester) and phenotypes were analyzed by observing at least three independent strains generated by transformation. To examine cell viability on agar plates, Phloxin B (SIGMA) was added to the media to a final concentration of 5mg/l.

Antibody Generation

The His$_{6}$-Hrs1 was expressed at 15°C and cell extracts were bound to Ni Sepharose 6 Fast Flow beads (GE Healthcare) and eluted with the Elution-U buffer (50 mM Tris pH 7.5, 500 mM NaCl, 5% Glycerol, 250 mM Imidazole pH 8.0, 0.5 mM DTT, 8 M Urea) followed by dialysis with D1 buffer (50 mM Tris pH 7.5, 500 mM NaCl, 5 % Glycerol, 150 mM Imidazole pH 8.0, 0.5 mM DTT, 4 M Urea). 3 mg of the recombinant Hrs1 was used to immunize rabbits (Cambridge Research Biochemicals). Anti-Hrs1 antisera (4030 final bleed) was used for subsequent experiments.

Western Blotting and Immunoprecipitation

To avoid protein degradation, PMSF (final 1mM) was added to the cell culture at the culturing temperature 1 min before harvesting. Cell pellets were frozen in liquid nitrogen and were kept at -80°C until required as previously described [1].

Immunoporecipitation of Hrs1-2xFLAG-GFP was done from native cell extracts. 400ul of JK buffer (50mM Tris-Cl (pH 7.8), 100mM NaCl, 5mM EGTA, 1mM EDTA, 1% TritonX-100, 1mM PMSF, 7.5% glycerol, 1 tablet/ml Complete mini (Roche)) [10] was added to the frozen cell pellets of 1.5 x 10^{8} cells. 500ul of acid washed glass beads (SIGMA) were added and cells were disrupted by

Fastprep24 (MP Biomedicals) at speed 6.5, 20 sec x 2 (20sec interval). Then NaCl was further added to a final concentration of 175mM. Supernatant of the soluble cell extracts were collected and 180ul of them were used for immunoprecipitation with 5 μg of the anti-FLAG anbitody (SIGMA) crross-linked to 25ul of the Dynabeads Protein G (invitrogen). After 45 min incubation on ice, the immunoprecipitates were washed 3 times with the JK buffer and were analyzed by the SDS-PAGE. λ-Phosphatase treatment was done on the immunoprecipitated Hrs1.T16A-2xFLAG-GFP. The immuno-complex was washed once with λ -PPase buffer (50mM Tris-HCl pH7.5, 0.1M NaCl, 0.1mM EGTA, 2mM DTT, 0.01% (w/v) Brij35, 15mM p-nitrophenyl phosphate (p-NPP)) and resuspended in 300ul of the λ-PPase buffer. 1 ul of λ -PPase (NEB, 400units/ul) was added to the reaction in the presence of 2mM $MnCl_2$ and incubated for 20min at 30°C. As negative controls, an inhibitor cocktail (10mM EGTA pH8.0, 0.1mM Na_2VO_3, 20mM b-glycerophosphate) or λ-PPase heat-inactivated at 65°C for one hour in the presence of 50mM EDTA was used.

Whole cell extracts were prepared as follows. RIPA buffer (10mM Na-Phosphate, pH 7.0, 1% Triton X-100, 0.1% SDS, 2mM EDTA, 150mM NaCl, 50mM NaF, 1mM PMSF, one tablet of Complete (Roche) / 10mL) was added to the frozen cell pellets and cells were boiled for 5min. The cells were then disrupted with acid washed glass beads (SIGMA) employing Fastprep24 (MP Biomedicals) at speed 6.5, 20 sec x 4. An equal volume of 3x Sample buffer (240 mM Tris-HCl (pH 6.8), 6 % SDS, 30 % Glycerol, 2.2 M β-mercaptoethanol, 0.05 % BPB) was added and the samples boiled for 5 min prior to analysis by SDS-PAGE. To detect α-tubulin, monoclonal antibody TAT-1, a generous gift from Keith Gull, was used at 1/2000 dilution. Anti-Hrs1 anti-sera (4030 final bleed) was used at 1/2000 to 1/8000 dilution to detect Hrs1 by Western blotting. To detect Hrs1-2xFLAG-GFP, anti-GFP monoclonal antibody (Roche) was used at 1/1000 dilution.

Detection of Ubiquitylation of Hrs1

A wildtype or *mts3.1* homothallic strain harbouring *hrs1-GFP-2xFLAG* was transformed with pRep1-6His-Ubi plasmid. The 6His-Ubi was induced for 15 hours at 25°C in MM+N medium. Cells were then induced for meiosis by firstly transferred to MM-N (1% glucose) for 7 hours at 25°C. Cells were then spun down and spotted on a SPA sporulation medium plate and incubated at 25°C for 6 hours before transferred to 34°C for 3.5 hours to partially inactivate the Mts3 function. Cells were then collected and cell extracts were prepared and purified by Ni-NTA beads as previously described [11]. Precipitated proteins were immunoblotted with anti-Ub (P4D1, Santa Cruz, sc-8017, 0.5μg/ml) and anti-Hrs1 4030 sera (1/1000).

Fluorescent Microscopy

Live imaging of the cells was conducted at 30°C using a Leica SP5 (objective : HCX PL APO Lbd. BI, 63x, NA1.4) and Olympus FV1000 (objective : UPlanSAPO, 60x, NA1.35) laser scanning confocal microscopes and a Nikon Eclipse Ti-E microscope (objective : Plan Apo VC, 100x, NA1.4) with an Andor iXon EM-DU897 camera and CoolLED pE-1 LED excitation system. Cells were immobilized on glass bottom dishes (MatTek Corporation) coated with lectin from Bandeiraea simplicifolia (SIGMA) and incubated with appropriate media. For each time point, images along the Z axis were taken every 0.5-0.6 mm to fully cover the thickness of the cell. Obtained images were processed by Huygens Essential (Scientific Volume Imaging), a deconvolution software, and maximum Z-projection images were generated by Image J (NIH).

Electron Microscopy, Tomography, and Immunolabelling

To obtain cells undergoing meiotic prophase with high synchronicity, KT2486 (*h^-/h^- pat1.114/pat1.114 lys1.131::mat-Pc-lys1$^+$ leu1.32/leu1.32 ade6-M210/ade6-M216*) and KT2919 (*h^-/h^- pat1.114/pat1.114 lys1.131::mat-Pc-lys1$^+$ leu1.32/leu1.32 ade6-M210/ade6-M216 hrs1::Hyg^r/hrs1::Hyg^r*) were generated and the culture was arrested at G1 at the permissive 25°C as

described above. The culture was then diluted with an equal volume of fresh MM-N (1% glucose, 50 mg/ml leucine), prewarmed at 34°C, and synchronous meiosis was induced by further incubating it at 34°C. A small portion of the culture was collected every hour to monitor the synchronicity. Cells for EM analysis were collected in a time series every 20 min from 0 min to 180min after the temperature shift and were cryoimmobilized by high-pressure freezing with Leica EMPACT-2. Freeze substitution of the cells was done using a freeze substitution device EM-AFS2 (Leica Microsystems, Vienna, Austria). The freeze substitution solution used for structural observations and tomography contained 0.2% glutaraldehyde, 0.2% uranyl acetate, 0.05% osmium and 1.25% water in acetone. For immunolocalisation studies a freeze substitution cocktail of 0.1% uranyl acetate and 1 % water dissolved in anhydrous acetone was used. The samples were substituted at -90°C for 50h. The temperature was then increased at a rate of 5°C/hour to -45°C followed by 4h incubation at -45°C. The samples were rinsed with acetone three times for 10 min each followed by lowicryl HM20 (Polysciences, Warrington, PA, USA) infiltration at -45°C with 25% lowicryl in acetone for 2h, 50% lowicryl for 2h and 75% lowicryl for 2h. The samples were then left in 100% lowicryl for 12h and in new 100% lowicryl for 2h before onset of polymerization. UV polymerization was applied for 48h at -45°C and then the temperature was increased to 20°C at a rate of 5°C/h. Finally the samples were left exposed to UV at room temperature for 48hr.

Sectioning was done on a Leica Ultra-cut UCT microtome (Leica Microsystems, Vienna, Austria) and serial sections were collected on Formvar-coated, palladium-copper slot grids.

For tomography Protein A conjugated with 15 nm gold (CMC university medical center Utrecht, Netherlands) was applied to both sides of the grid as fiducial markers. The grids were placed in a high-tilt holder (Model 2020; Fischione Instruments; Corporate Circle, PA) and the cells were recorded on a Tecnai F30 EM (FEI, Eindhoven, The Netherlands) operating at 300kV using the SerialEM software package (Mastronarde 2005). Images were taken every degree over a ±60° range on an FEI Eagle 4K x 4K CCD camera at a magnification of 20000x and a binning of 2 (pixel size 1.179 nm). Some SPBs were recorded as dual axis tilt series and some as single axis. The tilted images were aligned by using the positions of the fiducal gold particles, in the case of single axis tilt series patch tracking was used. The tomograms were generated using the R-weighted back-projection algorithm. To reconstruct complete SPBs, tomograms were collected from 3 serial sections, aligned and joined by using the eTomo graphical user interphase (Hoog et al, 2007; Ladinsky et al, 1994; Marsh et al, 2001; O'Toole et al, 2003). Tomograms were displayed as slices one voxel thick, modeled, and analyzed with the IMOD software package (Kremer et al, 1996). The morphology of MT ends were analyzed and classified as described in Hoog et al 2007. 3D distances between MTs and SPB or MT ends and SPB were measured with the MT kissing (mtk) program (Marsh et al., 2001).

The MT organisation at the SPB was evaluated by analysing the distribution of angles between MTs and the long axis of the SPB. The long axis of the SPB, here defined as the axis between the 2 lamellae of the SPB, was used as the reference vector ($\overrightarrow{AB}$). The MT vectors ($\overrightarrow{CD}$) were determined using the start and end points of each MT. The angle between the vectors was then calculated as follows:

$$\propto = \cos^{-1}\frac{\overrightarrow{AB}.\overrightarrow{CD}}{\|\overrightarrow{AB}\|.\|\overrightarrow{CD}\|} \propto = \cos^{-1}\frac{\overrightarrow{AB}.\overrightarrow{CD}}{\|\overrightarrow{AB}\|.\|\overrightarrow{CD}\|}.$$

The calculated angels are presented in 0-90 degree range in Fig. 1E by applying operations as follows: (i) 0 to 90 degree : no additional operation applied, (ii) 90 to 180 degree : subtract 180 and remove the "-" (convert the value to a positive value), (iii) 180 to 270 degree: subtract 180, (iv) 270 to 360 degree : subtract the value from 360.

For studies of SPB duplication through the time series, serial-sections (70 nm) through each entire SPB were studied and micrographs of each section was taken at the most appropriate stage tilt for visualizing the lamellae structure of the SPB. For each time point 10 or more SPBs were

studied. The SPBs were scored as doubled if 2 clear lamellae could be observed, if only one lamella could be seen it was scored single. In case of doubts they were labeled unknown.

Immunogold labelling was done by floating grids with serial sections (70 nm) on drops of blocking buffer consisting of 1.5% BSA and 0.1% fish skin gelatine in PBS for 30 min, followed by incubation on drops of Hrs1 antisera 4030, diluted 1:100, or anti γ-tubulin antibodies from mouse (product number T6557 from Sigma-Aldrich) diluted 1:5 antibodies in blocking buffer for 30 min, were rinsed off in 5 drops of PBS before the 10 nm gold conjugated Protein A (CMC university medical center Utrecht, Netherlands) was applied at a dilution of 1:70 for 20 min. For the γ-tubulin antibodies a linker rabbit anti mouse (DAKO) was applied at 1:150 dilution in blocking buffer for 20 min before applying Protein A. The labeling was followed by an extensive rinse of totally 1h on 10 drops of PBS. The sections were then fixed in 1% glutaraldehyde in PBS for 5 min before they were rinsed in 10 drops of water and post stained using uranyl acetate and lead citrate. The sections were viewed using a CM120 biotwin electron microscope (FEI, Eindhoven, The Netherlands) operating at 100 kV. Digital acquisitions were made with a Keen View CCD camera (Soft Imaging System, Muenster, Germany). Series of sections through complete SPBs was observed to determine the localization of Hrs1. For each experiment 10 SPBs or more were examined. For meiotic *S.pombe* the cells from time point 120 min after induction of meiosis were used for both *hrs1*$^{+}$ and *hrs1*Δ. Models of immunolabelling were done by joining serial sections in the IMOD software package (Kremer et al, 1996).

Quantitative PCR (qPCR)

Total RNA was prepared as previously described [12] from cells undergoing synchronous meiosis. RNA quality was examined by an Agilent Bioanalyser. A reverse transcriptional reaction was performed using SMARTScribe Reverse Transcriptase (Clontech) and oligo dT primers. The qPCR reaction was set up employing the Maxima SYBR Green/ROX qPCR Master Mix (Fermentas). The reactions and analysis were carried out employing a LightCycler 480 (Roche).

To amplify the *hrs1* cDNA, primers hrs1-1F (5' CTCCATTGTTGGACGTTCACTC) and hrs1-1R (5' CGGCGAAGGTCTTCTGTAATCT) were used. To amplify the *act1* cDNA, primers act1-2F (5' TTCCTCATGCTATCATGCGTCT) and act1-2R (5' GCGACGTAGCAAAGTTTCTCCT) were used. The *hrs1* expression level was normalized against *act1* expression level.

Liquid Chromatography and Tandem Mass Spectrometry (LC-MS/MS)

Cell Extract Preparation

To identify the phosphorylation sites of Hrs1, synchronous meiotic cell lysates were prepared from KT2474 (*h*$^{-}$/*h*$^{-}$ *pat1.114/pat1.114 ade6-M216/ade6-M210 leu1/leu1 lys1::mat-Pc-lys1*$^{+}$ *hrs1.TA-2xFLAG-GFP::Kan*^{r}/*hrs1.TA-2xFLAG-GFP::Kan*^{r}). 4 L of culture, with a cell density of 8 x 10^6 cells/ml in YE (without adenine) medium, was filtrated and washed extensively with MM-N (1% glucose, 50 mg/ml leucine), then resuspended in 8 L of MM-N and cultured at 25°C for 6 hours 15 min. The culture was then diluted with 8L of fresh MM-N pre-warmed at 34°C, then incubated at 34°C for 135 min to synchronously induce transition of meiotic prophase to MI. 1 min before harvesting the cells, PMSF was added to a final concentration of 1mM. The culture was centrifuged and the cell pellet was washed with cdc2 STOP buffer[13] and resuspended in 16 ml of the JK buffer (50 mM Tris-Cl (pH 7.8), 100 mM NaCl, 5m M EGTA, 1 mM EDTA, 1% TritonX-100, 1 mM PMSF, 7.5% glycerol, 1 tablet/ml Complete mini (Roche))[10]. The thick cell suspension was dropped into liquid nitrogen to generate "popcorns" which were then ground using a SPEX SamplePrep freezer mill (No. 6870). The grinding cycle was 10 cycles of 2 min pre-cooling and 2 min grinding, impact rate 12. The ground cells were thawed on ice and 160 ml of JK buffer added and NaCl added to a final concentration of 175 mM. Cell extracts were spun down and 130 ml of the supernatant was taken for immunoprecipitation with 180 mg of anti-FLAG antibody (SIGMA) cross-liked to 900 μl of Dynabeads

(Invitrogen). The sample was then incubated on ice for 90 min and washed twice with 10 ml of JK buffer. The Dynabeads were then resuspended in NuPAGE loading mixture (1 x NuPAGE LDS Sample Buffer (Invitrogen), 1 X NuPAGE Reducing Agent (Invitrogen) in JK buffer) and loaded on a 4-12% gradient gel (Bis-Tris Mini Gel, 1 mm, Invitrogen). The gel was fixed in 7% glacial acetic acid in 40% (v/v) methanol for one hour before stained with Brilliant Blue G-Colloidal Cencentrate (SIGMA) (diluted to 80% with methanol) for one hour. The gel was then destained with 10% acetic acid in 25% (v/v) methanol for 60 seconds followed by 25% methanol. The Hrs1.TA-2xFLAG-GFP band was excised and subjected to LC-MS/MS analysis.

Gel Band Destaining and Washing

Coomassie blue stained gel bands were destained with 3 x 20 minute changes of 1ml 200 mM ammonium bicorbante, 40% (V/V) acetonitrile. Gel bands were then dehydrated by the addition of 500 µl acetonitrile for 15 minutes followed by rehydration in 500 µl of water for a further 15 minutes. This dehydration-rehydration procedure was performed a total of three times followed by a final dehydration in acetonitrile.

In-Gel Tryptic Digestion

Gel bands were rehydrated in 25 µl of 50 mM ammonium bicarbonate, 9% (v/v) acetonitrile, 20 ng/µl sequencing grade trypsin (Sigma-Aldrich) for 20 minutes. The bands were then covered in 100 µl of 50 mM ammonium bicarbonate, 9% (v/v) acetonitrile and incubated at 37°C for 18hrs. Following digestion, samples were acidified by the addition on 10 µl of 10% (v/v) formic acid. The digest supernatant was then transferred to a fresh Eppendorf tube and dried in a vacuum centrifuge at 40°C for 30 minutes. The dried peptides were then resuspended in 20µl of water, 0.1% trifluoroacetic acid (Sigma-Aldrich) prior to LC-MS/MS analysis.

nLCMSMS Analysis

Peptides were separated utilizing a Nano-Acquity UPLC system (Waters) as detailed below. Samples were loaded onto a Waters C18 Symmetry trap column (180 µm ID, 5 µm 5 cm) in water, 0.1% (v/v) acetonitrile, 0.1% (v/v) formic acid at a flow rate of 7µl/min for 5 minutes. Peptides were then separated using a Waters NanoAcquity BEH C18 column (75 um ID, 1.7um, 25cm) with a gradient of 1 to 25% (v/v) of acetonitrile, 0.1% formic acid over 30 minutes at a flow rate of 400 nl/min.

The nLC effluent was sprayed directly into the LTQ-Orbitrap XL mass spectrometer aided by the Proxeon nano source at a voltage offset of 1.8 KV. The mass spectrometer was operated in parallel data dependent mode where the MS survey scan was performed at a nominal resolution of 60, 000 (at m/z 400) resolution in the Orbitrap analyzer between m/z range of 400-2000. The top 6 multiply charged precusors were selected for CID in the LTQ at normalized collision energy of 35% with multistage activation enabled at neutral losses of 49.0, 32.7 and 24.5 Da. Dynamic exclusion was enabled to prevent the selection of a formally targeted ion for a total of 20 seconds.

Two Hybrid Assays

Two-hybrid assays of Hrs1 and interacting proteins were carried out as previously described[1]. Full-length *kms1* cloned in pACT2 was a generous gift from Osami Niwa[14].

Author Contributions

C.F., S.S., S.P., M.O., Y.C., J.M., H.M., A.G., D.L.S. and C.A. designed and performed the experiment and analyzed the data. M.Y. provided reagents and strains. K.T. planned the project, designed and performed the experiment, analyzed the data and wrote the paper with input from the co-authors.

Supplemental Information

1. Tanaka, K., Kohda, T., Yamashita, A., Nonaka, N., and Yamamoto, M. (2005). Hrs1p/Mcp6p on the meiotic SPB organizes astral microtubule arrays for oscillatory nuclear movement. Curr Biol *15*, 1479-1486.
2. Nabeshima, K., Nakagawa, T., Straight, A.F., Murray, A., Chikashige, Y., Yamashita, Y.M., Hiraoka, Y., and Yanagida, M. (1998). Dynamics of centromeres during metaphase-anaphase transition in fission yeast: Dis1 is implicated in force balance in metaphase bipolar spindle. Mol Biol Cell *9*, 3211-3225.
3. Yamamoto, A., and Hiraoka, Y. (2003). Monopolar spindle attachment of sister chromatids is ensured by two distinct mechanisms at the first meiotic division in fission yeast. Embo J *22*, 2284-2296.
4. Bahler, J., Wu, J.Q., Longtine, M.S., Shah, N.G., McKenzie, A., 3rd, Steever, A.B., Wach, A., Philippsen, P., and Pringle, J.R. (1998). Heterologous modules for efficient and versatile PCR-based gene targeting in Schizosaccharomyces pombe. Yeast *14*, 943-951.
5. Wach, A. (1996). PCR-synthesis of marker cassettes with long flanking homology regions for gene disruptions in S. cerevisiae. Yeast *12*, 259-265.
6. Sato, M., Dhut, S., and Toda, T. (2005). New drug-resistant cassettes for gene disruption and epitope tagging in Schizosaccharomyces pombe. Yeast *22*, 583-591.
7. Hopp, T.P., Prickett, K.S., Price, V.L., Libby, R.T., March, C.J., Ceretti, D.P., Urdal, D.L., and Conlon, P.J. (1988). A Short Polypeptide Marker Sequence useful for Recombinant Protein Identification and Purification. Nature Biotechnology *6*, 1204-1210.
8. Shaner, N.C., Campbell, R.E., Steinbach, P.A., Giepmans, B.N., Palmer, A.E., and Tsien, R.Y. (2004). Improved monomeric red, orange and yellow fluorescent proteins derived from Discosoma sp. red fluorescent protein. Nat Biotechnol *22*, 1567-1572.
9. Higuchi, R., Krummel, B., and Saiki, R.K. (1988). A general method of in vitro preparation and specific mutagenesis of DNA fragments: study of protein and DNA interactions. Nucleic Acids Res *16*, 7351-7367.
10. Wigge, P.A., and Kilmartin, J.V. (2001). The Ndc80p complex from Saccharomyces cerevisiae contains conserved centromere components and has a function in chromosome segregation. J Cell Biol *152*, 349-360.
11. Shiozaki, K., and Russell, P. (1997). Stress-activated protein kinase pathway in cell cycle control of fission yeast. Methods Enzymol *283*, 506-520.
12. Lyne, R., Burns, G., Mata, J., Penkett, C.J., Rustici, G., Chen, D., Langford, C., Vetrie, D., and Bahler, J. (2003). Whole-genome microarrays of fission yeast: characteristics, accuracy, reproducibility, and processing of array data. BMC Genomics *4*, 27.
13. Moreno, S., Klar, A., and Nurse, P. (1991). Molecular genetic analysis of fission yeast Schizosaccharomyces pombe. Methods Enzymol *194*, 795-823.
14. Miki, F., Kurabayashi, A., Tange, Y., Okazaki, K., Shimanuki, M., and Niwa, O. (2004). Two-hybrid search for proteins that interact with Sad1 and Kms1, two membrane-bound components of the spindle pole body in fission yeast. Mol Genet Genomics *270*, 449-461.
15. Lupas, A., Van Dyke, M., and Stock, J. (1991). Predicting coiled coils from protein sequences. Science *252*, 1162-1164.
16. West, R.R., Vaisberg, E.V., Ding, R., Nurse, P., and McIntosh, J.R. (1998). cut11(+): A gene required for cell cycle-dependent spindle pole body anchoring in the nuclear envelope and bipolar spindle formation in Schizosaccharomyces pombe. Mol Biol Cell *9*, 2839-2855.
17. Thompson, J.D., Higgins, D.G., and Gibson, T.J. (1994). CLUSTAL W: improving the sensitivity of progressive multiple sequence alignment through sequence weighting, position-specific gap penalties and weight matrix choice. Nucleic Acids Res *22*, 4673-4680.
