# Supplementary figures and images for: Transient Structure Associated with the Spindle Pole Body Directs Meiotic Microtubule Reorganization in S. pombe
Source: Curr Biol. 2012 Apr 10;22(7):562–74. doi: 10.1016/j.cub.2012.02.042 (PMC3382715; doi:10.1016/j.cub.2012.02.042)

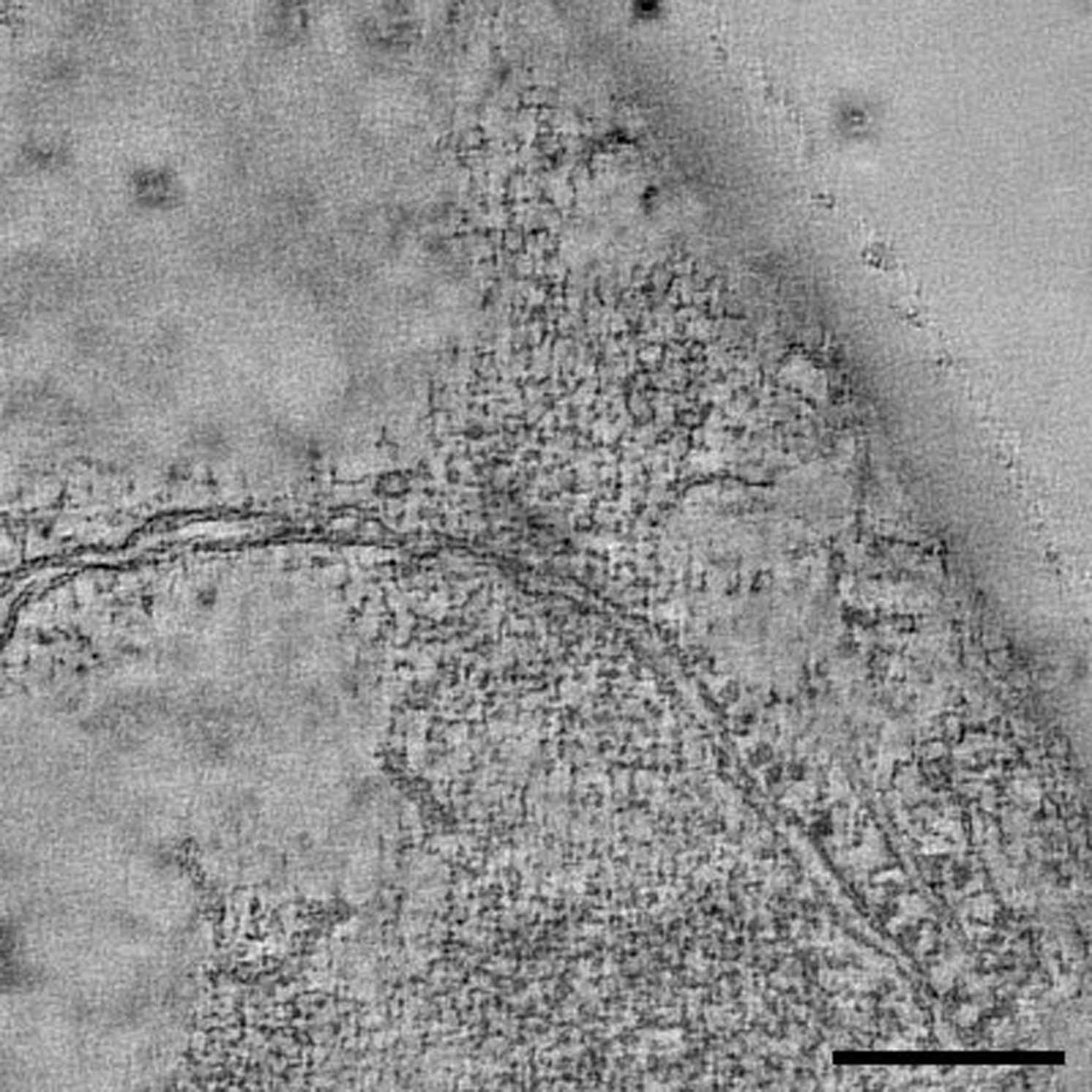

Supplement: Movie S1. Tomogram of WT Strain, Related to Figure 1 — WT cells from 80 min after inducing synchronous meiosis. A movie through the 250 nm thick section, which is the middle section from the three sections used for modeling in Figure 1 and Movie S3, is shown. It harbors the complete SPB. Note the two lamellar structures (a duplicated SPB) and that the microtubules are gathering at the SPB from different directions. Some MTs are longitudinally sectioned and some are cross-sectioned. Scale bar represents 200 nm. [file mmc2.jpg]

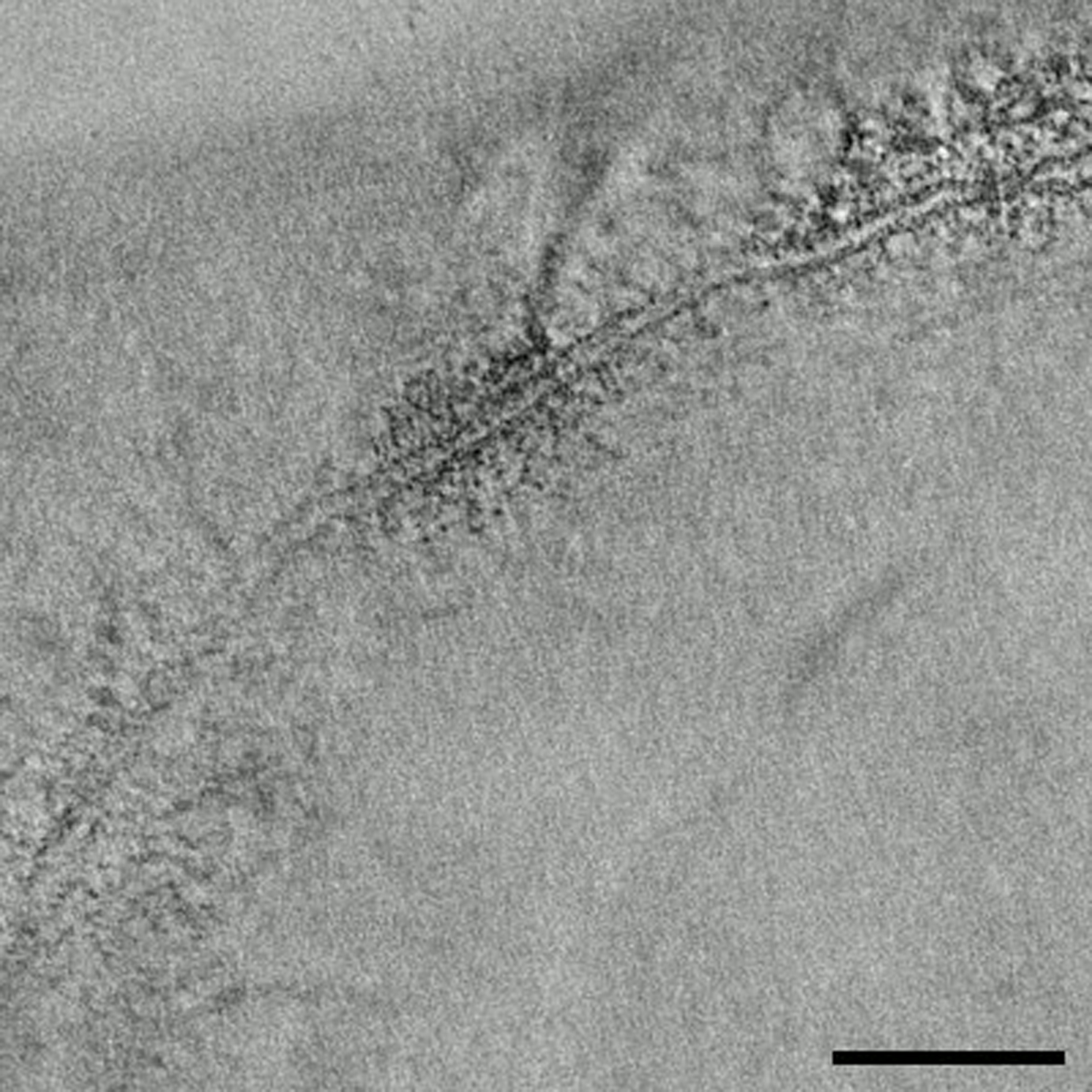

Supplement: Movie S2. Tomogram of hrs1 Deletion Strain, Related to Figure 1 — Cells of hrs1 deletion strain from 80 min after inducing synchronous meiosis. A movie through the 250 nm thick section, which is the middle section from the three sections used for modeling in Figure 1 and in Movie S4, is shown. It harbors the complete SPB. Note the two lamellar structures (a duplicated SPB) and that the MTs are parallel and longitudinally sectioned. Scale bar represents 200 nm. [file mmc3.jpg]

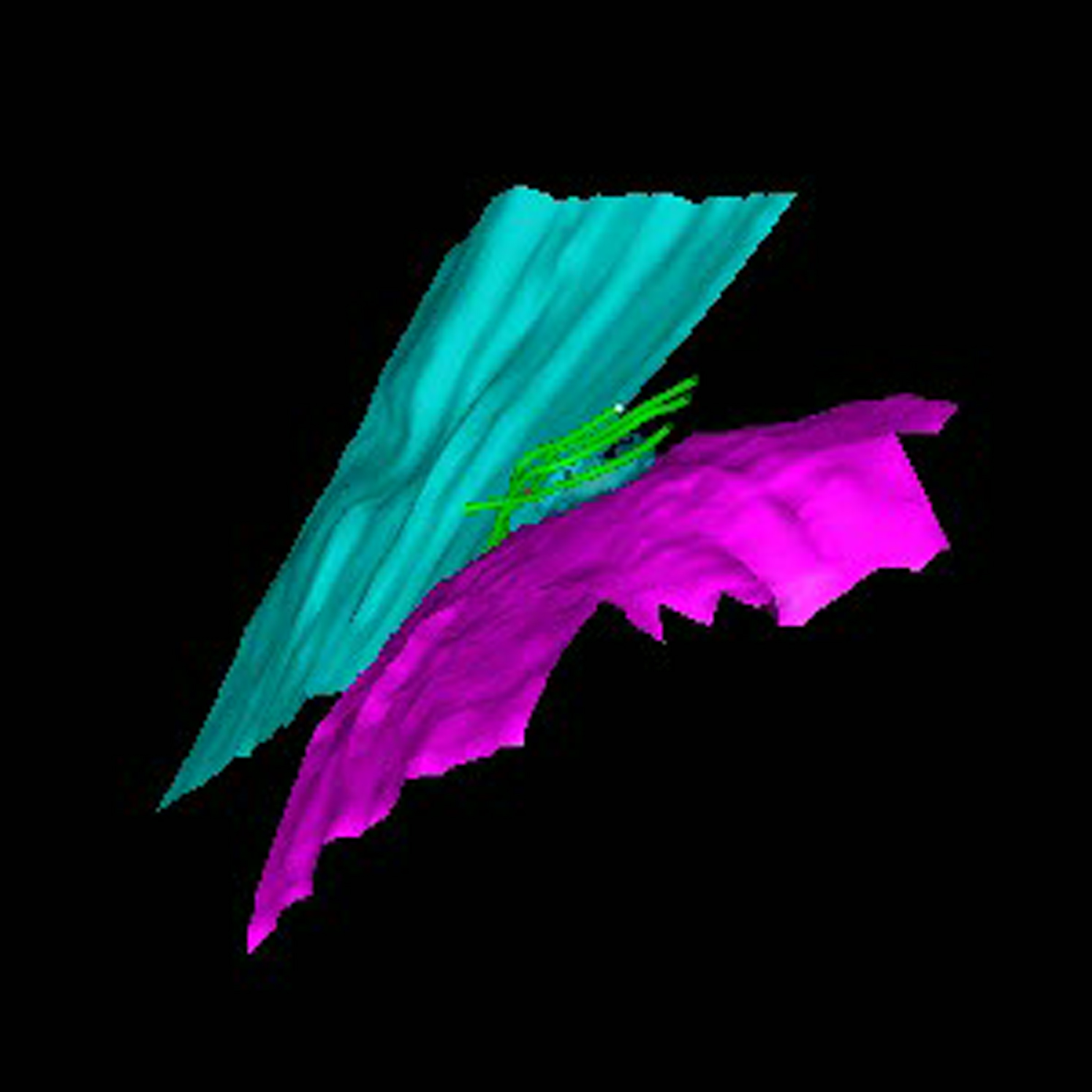

Supplement: Movie S4. Model of hrs1 Deletion Strain, Related to Figure 1 — A model from the SPB from a hrs1 deletion cell 80 min after inducing synchronous meiosis was made from three consecutive sections of 250 nm thickness. The MTs (green) are parallel and pass close to the SPB (light blue), which also have two lamellar structures (black). The nuclear envelope is shown in purple and the plasma membrane in turquoise. The MT ends have been identified and are presented by colors. Capped ends (red), blunt ends (blue), sheet (yellow), and undetermined ends are shown in white. [file mmc5.jpg]
